# Deficiency of Inositol Monophosphatase Activity Decreases Phosphoinositide Lipids and Enhances TRPV1 Function *In Vivo*

**DOI:** 10.1523/JNEUROSCI.0803-20.2020

**Published:** 2021-01-20

**Authors:** Rebeca Caires, Briar Bell, Jungsoo Lee, Luis O. Romero, Valeria Vásquez, Julio F. Cordero-Morales

**Affiliations:** ^1^Department of Physiology, College of Medicine, University of Tennessee Health Science Center, Memphis, Tennessee 38163; ^2^Integrated Biomedical Sciences Graduate Program, College of Graduate Health Sciences, University of Tennessee Health Science Center, Memphis, Tennessee 38163

**Keywords:** *Caenorhabditis elegans*, *in vivo* calcium imaging, inositol monophosphatase, lipid regulation, phosphoinositides, TRPV1

## Abstract

Membrane remodeling by inflammatory mediators influences the function of sensory ion channels. The capsaicin- and heat-activated transient receptor potential vanilloid 1 (TRPV1) channel contributes to neurogenic inflammation and pain hypersensitivity, in part because of its potentiation downstream of phospholipase C-coupled receptors that regulate phosphoinositide lipid content. Here, we determined the effect of phosphoinositide lipids on TRPV1 function by combining genetic dissection, diet supplementation, and behavioral, biochemical, and functional analyses in *Caenorhabditis elegans*. As capsaicin elicits heat and pain sensations in mammals, transgenic TRPV1 worms exhibit an aversive response to capsaicin. TRPV1 worms with low levels of phosphoinositide lipids display an enhanced response to capsaicin, whereas phosphoinositide lipid supplementation reduces TRPV1-mediated responses. A worm carrying a TRPV1 construct lacking the distal C-terminal domain features an enhanced response to capsaicin, independent of the phosphoinositide lipid content. Our results demonstrate that TRPV1 activity is enhanced when the phosphoinositide lipid content is reduced, and the C-terminal domain is key to determining agonist response *in vivo*.

## Abstract (precis)

Membrane remodeling by inflammatory mediators influences the function of sensory ion channels. The capsaicin- and heat-activated transient receptor potential vanilloid 1 (TRPV1) channel contributes to neurogenic inflammation and pain hypersensitivity, in part because of its potentiation downstream of phospholipase C-coupled receptors that regulate phosphoinositide lipid content.

## Significance Statement

Transient receptor potential vanilloid 1 (TRPV1) is an essential protein for the mechanism whereby noxious stimuli, such as high temperatures and chemicals, cause pain. TRPV1 undergoes sensitization, a process in which inflammatory molecules enhance its response to other stimuli, thereby promoting pain hypersensitivity. Proalgesic agents produced in response to tissue injury activate phospholipase C-coupled receptors and alter the membrane phosphoinositide lipid content. The mechanism by which phosphoinositide lipids modulate TRPV1 function has remained controversial. Determining whether membrane phosphoinositides are positive or negative regulators of TRPV1 function is critical for developing therapeutic strategies to ameliorate TRPV1-mediated inflammatory pain. We address the role of phosphoinositide lipids on TRPV1 function using an *in vivo* approach and report that phosphoinositide lipids reduce TRPV1 activity *in vivo*.

## Introduction

The transient receptor potential vanilloid 1 (TRPV1) is a polymodal ion channel activated by noxious heat (Caterina et al., 1997), capsaicin (Caterina et al., 1997), animal toxins (Siemens et al., 2006; Bohlen et al., 2010; Yang et al., 2015), extracellular protons (Jordt et al., 2000), and lysophosphatidic acid (Nieto-Posadas et al., 2011). TRPV1 is modulated by proalgesic inflammatory agents, such as bradykinin (Chuang et al., 2001), nerve growth factor (Lewin et al., 1993; Chuang et al., 2001; Stratiievska et al., 2018), and bioactive lipids (Zygmunt et al., 1999; Prescott and Julius, 2003; Cao et al., 2013b) produced in response to tissue injury. TRPV1 is expressed in nociceptors, which are specialized peripheral sensory neurons that detect and respond to a variety of potentially harmful stimuli, including extreme temperatures, pressure, and chemicals (Basbaum et al., 2009). TRPV1 undergoes sensitization, a process in which inflammatory agents enhance its response to other stimuli, thereby promoting pain hypersensitivity (Basbaum et al., 2009). Sensitization is an intrinsic feature of TRPV1 since protons (e.g., pH 6), fatty acids [e.g., arachidonic acid (AA)], and the lack of phosphoinositide lipids produce a leftward shift in the thermal and chemical response profile of TRPV1-containing liposomes (Cao et al., 2013b). However, TRPV1 is also indirectly sensitized through the activation of phospholipase C (PLC)-coupled receptors by a variety of proalgesic agents (e.g., bradykinin) and the subsequent hydrolysis of phosphatidylinositol 4,5-bisphosphate (PIP_2_; Chuang et al., 2001) and/or phosphorylation via protein kinase C (Vellani et al., 2001; Numazaki et al., 2002; Bhave et al., 2003). It has been reported that reduction of plasma membrane PIP_2_, via PLC-mediated hydrolysis, sensitizes TRPV1, mimicking the effect observed with proalgesic agents such as bradykinin and nerve growth factor (Chuang et al., 2001; Prescott and Julius, 2003). However, later work demonstrated that TRPV1 is also modulated by other phosphoinositide lipids, including PI, PI3P, PI4P, and PI5P (Lukacs et al., 2007; Klein et al., 2008; Cao et al., 2013b). Hence, particular attention has been given to the mechanism by which this lipid class modulates channel function (Rohacs, 2015). Defining the mechanisms through which bioactive lipids modulate TRPV1 will help to elucidate its role in controlling sensory neuron excitability.

Several lines of evidence are consistent with the idea that phosphoinositides negatively regulate channel function. At low capsaicin concentration and moderate heat, it has been shown that a soluble PIP_2_ partially inhibits TRPV1 in a whole-cell environment (Lukacs et al., 2007). Positively charged residues (lysine/arginine) within the distal TRPV1 C-terminal domain (L777–L792 in rat TRPV1) have been identified as the site required for phosphoinositide lipid-mediated inhibition, and mutations that eliminate these residues enhance channel heat response (Prescott and Julius, 2003). Interestingly, a vampire bat TRPV1 splice variant lacking the distal C-terminal domain (putative phosphoinositide interaction site) displayed enhanced sensitivity to thermal stimuli (Gracheva et al., 2011). These results are further supported by experiments in which TRPV1 displays enhanced sensitivity to thermal and chemical stimuli when reconstituted into liposomes without phosphoinositide lipids (Cao et al., 2013b). Furthermore, incorporation of phosphoinositide lipids (e.g., PI, PI3P, PI4P, PI5P, and PIP_2_) in the proteoliposomes produces a rightward shift (i.e., decreased response) in the agonist response profile of the channel. Likewise, stabilization of the TRPV1 C terminus–membrane interaction, using nitrilotriacetic acid (NTA)-modified lipid (DGS (1,2-dioleoyl-sn-glycero-3)-NTA) in a nickel-dependent manner, displays a TRPV1 agonist response comparable to those observed in the presence of phosphoinositide lipids (Cao et al., 2013b). Moreover, it has also been shown that phosphoinositide turnover enhances TRPV1 function mediated by AKAP150 (a kinase anchor protein; Jeske et al., 2011). Together, this experimental evidence supports a model whereby phosphoinositide lipids decrease TRPV1 function.

This model, however, has been challenged by studies supporting phosphoinositides as positive modulators of TRPV1 activity (Rohacs, 2015). For instance, previous work has shown that TRPV1 activity is inhibited by phosphoinositide sequestration and potentiated by direct application of a soluble synthetic short-chain phosphatidylinositol 4,5-bisphosphate dioctanoyl (DiC8-PIP_2_; Stein et al., 2006; Kim et al., 2008b; Poblete et al., 2015; Sun and Zakharian, 2015). Chimera analyses swapping the proximal C-terminal domains of TRPV1 and TRPM8 supported the existence of an activation domain for PIP_2_ (Brauchi et al., 2007). Using lipid phosphatases, Klein et al. (2008) demonstrated that the depletion of phosphoinositides inhibits TRPV1 activation by capsaicin. Experiments using a *Pirt* (i.e., a phosphoinositide interacting regulator of TRP) knock-out mouse support the notion that *Pirt* and PIP_2_ are both required to enhance TRPV1 function (Kim et al., 2008a). Patch-clamp and fluorescence binding assays with a phosphoinositide analog suggest that the proximal C-terminal region (S711–P732 in rat TRPV1) of TRPV1 interacts with phosphoinositides and, in turn, potentiates channel function (Ufret-Vincenty et al., 2011). Likewise, mutagenesis experiments, combined with a voltage-sensitive phosphatase, also support a positive role for the interaction between the proximal C-terminal region of TRPV1 and phosphoinositides (Ufret-Vincenty et al., 2015). Previous work using purified TRPV1 incorporated into planar lipid bilayers showed that DiC8-PIP_2_ is a positive cofactor for its activity (Lukacs et al., 2013a; Sun and Zakharian, 2015). Interestingly, Senning et al. (2014) found potentiation of TRPV1 activity when DiC8-PIP_2_ was added to the intracellular leaflet, whereas inhibition was observed when added to both leaflets of the membrane. On the other hand, a dual regulatory effect of phosphoinositide lipids on TRPV1 activity (depending on the stimuli concentration) was shown by coexpressing the channel with a phosphatidylinositol-4-phosphate 5-kinase (Lukacs et al., 2007, 2013b). Thus, by using a variety of experimental approaches, it has been shown that TRPV1 can be either positively or negatively regulated by phosphoinositide lipids. Accordingly, a consensus view has not emerged regarding the mechanisms by which phosphoinositide turnover modulates TRPV1 function.

Genetic manipulation of phosphoinositide lipid content has been challenging because these bioactive lipids are ubiquitous and essential for a variety of physiological processes. In *Caenorhabditis elegans*, phosphatidylinositol (PI) can be synthesized downstream of the dephosphorylation of inositol monophosphate (IP1) by the myo-inositol monophosphatase (IMPase) (Kimata et al., 2012). Importantly, a C. elegans strain lacking the function of the inositol monophosphatase enzyme (TTX-7; IMPase worm ortholog) reaches adulthood with mild phenotypes. Previous work inferred that the absence of the TTX-7 enzyme, or its inhibition by LiCl, generates worms with reduced levels of phosphoinositide lipids (Tanizawa et al., 2006; Kimata et al., 2012). Rat TRPV1 channels expressed in ASH neurons of worms elicit capsaicin-aversive behavior, similar to the functional and pharmacological features observed in mammalian neurons (Tobin et al., 2002; Liedtke et al., 2003; Kahn-Kirby et al., 2004; Caires et al., 2017; Geron et al., 2018). Notably, the TTX-7 enzyme is expressed in ASH neurons (Tanizawa et al., 2006). Although the mammalian TRPV1 native membrane environment might be different from that of ASH neurons, TRPV1 features similar functional properties (e.g., polymodal activation and modulation) when measured in cells or systems with different lipidic composition (e.g., cultured dorsal root ganglia neurons, human embryonic kidney (HEK293) cells, *Xenopus* oocytes, insect cells, soybean lipids, or synthetic liposomes; Caterina et al., 1997; Nieto-Posadas et al., 2011; Rivera-Acevedo et al., 2013; Cao et al., 2013b; Jara-Oseguera et al., 2016). Based on this knowledge, we sought to gain insight into TRPV1 regulation by phosphoinositide lipids by genetically manipulating their content in worms.

Here, by combining genetic dissection, diet supplementation, and behavioral, biochemical, and functional assays with Ca^2+^ imaging, we demonstrate that TRPV1 activity is enhanced when phosphoinositide lipid content is reduced. As in mammalian cells, the aversive response of transgenic TRPV1 worms can be positively modulated by AA and by a tarantula toxin [Double Knot Toxin (DkTx)], while also being negatively modulated by capsazepine (a TRPV1 antagonist). Chemical inhibition of the TTX-7 enzyme of the worms with LiCl enhances channel function *in vivo*. TRPV1 worms lacking the function of the TTX-7 enzyme have decreased levels of phosphoinositide lipids and display enhanced aversive behavior to capsaicin. Conversely, worms supplemented with phosphoinositide lipids in their diet display reduced aversive behavior. Live-worm imaging in TRPV1-expressing neurons shows that capsaicin elicits robust Ca^2+^ transients in worms fed with the control diet or lacking the function of TTX-7, but also shows significantly less Ca^2+^ influx in worms grown with a phosphoinositide lipid-supplemented diet. Behavioral and Ca^2+^-imaging analyses of a worm carrying a TRPV1 construct lacking the distal C-terminus phosphoinositide interaction site feature enhanced response to capsaicin, independent of the phosphoinositide lipid content, thus supporting that this site within the distal C-terminal domain is a key region in determining agonist response. Altogether, our results support that TRPV1 function *in vivo* is enhanced when phosphoinositide lipid content is reduced.

## Materials and Methods

### 

#### Strains

Worms were propagated as previously described (Brenner, 1974). Wild-type (WT; N2) and mutant IK589 *ttx-7(nj50) I* strains were obtained from the Caenorhabditis Genetics Center, which is funded by the National Institutes of Health Office of Research Infrastructure Programs (Grant P40-OD-010440). Using the MosSCI (Mos1-mediated single-copy insertion) method (Frøkjær-Jensen et al., 2012; Vásquez, 2020), we engineered transgenic TRPV1 worms expressing wild-type rat TRPV1 and Δ764-TRPV1 constructs at isogenic levels (i.e., under the same promoter to achieve similar expression), allowing us to compare behavior and function across different strains and diets. These strains are as follows: COP1493 *knuSi749 [pnu1336 (osm10p::rtrpv1-wt-optimized::tbb-2u, unc-119(+))] II; unc119(ed3) III; osm-9(ky10) IV; Posm-10::GFP X*; and COP1503 *knuSi752 [pnu1336 (osm10p::rtrpv1-Δ764-optimized::tbb-2u, unc-119(+))] II; unc-119(ed3) III; osm-9(ky10) IV; Posm-10::GFP X*. Strains were crossed to obtain the following: VVR32 *ttx-7(nj50) I; kyls200 X = elt-2::gfp + Psra6::rtrpv1cDNA*, VVR47 *ttx-7(nj50) I; knuSi749[pnu1336(Posm-10::rtrpv1-wt-optimized::3'UTR tbb-2, unc-119(+))] II*, and VVR52 *knuSi749 [pnu1336 (osm10p::rtrpv1-wt-optimized::tbb-2u, unc-119(+))] II; unc119(ed3) III*. For Ca^2+^-imaging assays, we crossed TRPV1 (wild-type or Δ764) and *ttx-7*; TRPV1 worms with worm CX6632 carrying GCaMP (a genetically encoded Ca^2+^ indicator) in an extrachromosomal array. CX6632 was donated by the Bargmann laboratory (Kahn-Kirby et al., 2004). The GCaMP gene encodes a fusion protein of EGFP, calmodulin, and the M13 peptide from myosin light-chain kinase. The *sra-6* promoter was used to drive the expression of GCaMP in ASH neurons. GCaMP was initially obtained from vector pN1-G-CaMP (catalog #RDB06747, RIKEN BioResource Center). These GCaMP carrier strains are as follows: VVR54 *knuSi749 [pnu1336 (osm10p::rtrpv1-wt-optimized::tbb-2u, unc-119(+))] II; unc119(ed3) III; osm-9(ky10) IV; kyEx728 [sra-6p::G-CaMP]*, VVR55 *ttx-7(nj50) I; knuSi749 [pnu1336 (osm10p::rtrpv1-wt-optimized::tbb-2u, unc-119(+))] II; unc119(ed3) III; osm-9(ky10) IV; kyEx728 [sra-6p::G-CaMP]*, and VVR57 *knuSi752 [pnu1336 (osm10p::rtrpv1- Δ 764-optimized::tbb-2u, unc-119(+))] II; unc-119(ed3) III; osm-9(ky10) IV; kyEx728 [sra-6p::G-CaMP]*.

#### Behavioral assays

We placed worm eggs on plates seeded with OP50 (an *Escherichia coli* uracil auxotroph strain that is used as a food source for worms), with and without PIP_2_. Young adult worms on the plates (produced from seeded eggs after 3 d at 20°C) were rinsed with M13 buffer (30 mm Tris-HCl, 100 mm NaCl, 10 mm KCl, pH 7) and transferred to a plate without food, 15 min prior to the drop-behavioral assay (Hart, 2006).

##### Drop test

Behavioral trials were performed by placing a drop containing M13 buffer plus 1% v/v ethanol with or without various concentrations of capsaicin (Tocris Bioscience), glycerol (1 m), or CuCl_2_ (1 mm) in front of a moving young adult hermaphrodite worm as previously described (Hart, 2006).

##### Gentle-body and nose touch

Worms were tested for their ability to avoid mechanical stimuli as previously described (Hart, 2006; Geffeney et al., 2011; Vásquez et al., 2014). Withdrawal responses were scored as a dichotomous variable.

#### Diet supplementation

The nematode growth media (NGM) plates were supplemented with 0.1% TERGITOL, and with the following, when indicated: 0.2 mm AA (Nu-Chek Prep; Watts and Browse, 2002; Caires et al., 2017), 0.2 mm PIP_2_ (Avanti Polar Lipids), 0.1 mm capsazepine (Tocris Bioscience), or 7.5 mm LiCl.

#### Toxin incubation

Before the drop test assay, worms were incubated for 15 min with M13 buffer containing DkTx (5 μM; Geron et al., 2018; Vásquez, 2020).

#### Protein expression determination

We placed worm eggs on 10 cm plates seeded with OP50, with and without PIP_2_, and grew them at 20°C for 3 d. Young adult hermaphrodite worms were collected with M9 buffer (86 mm NaCl, 42 mm Na_2_HPO_4_, 22 mm KH_2_PO_4_, 1 mm MgSO_4_) and treated with the Mem-PER Plus Membrane Protein Extraction Kit (Thermo Fisher Scientific) as follows. Worms (∼9000) were washed twice with cold Cell Wash Solution (with Halt Protease Inhibitor Cocktail, Thermo Fisher Scientific). Pelleted worms were resuspended with 1 ml of cold Permeabilization Buffer (with Halt Protease Inhibitor Cocktail) and homogenized with a sonicator. An extra 1 ml of cold Permeabilization Buffer was added to the homogenate and incubated for 10 min with constant mixing at 4°C. Homogenates were spun down at 16,000 × *g* for 15 min at 4°C. Permeabilized cells were resuspended in 500 µl of cold Solubilization Buffer (with Halt Protease Inhibitor Cocktail) and incubated for 30 min at 4°C with constant mixing. Solubilized cells were spun down at 16,000 × *g* for 15 min at 4°C. Supernatants containing solubilized membrane proteins were transferred to a new tube. Protein concentration was measured with the Bio-Rad protein assay, and 30 µg of total protein was loaded in Mini-PROTEAN TGX Stain-Free Precast Gels (Bio-Rad). Mouse monoclonal anti-TRPV1 (1:1000; catalog #203103, Abcam) and HRP-conjugated goat anti-mouse secondary antibodies (1:2000; catalog #205719, Abcam) were used for western blots. Membranes were developed with SuperSignal West Femto Maximum Sensitivity Substrate (Thermo Fisher Scientific) and imaged in a ChemiDoc Touch Imaging System (Bio-Rad) for chemiluminescence. Western blots were analyzed using Image Lab Software (Bio-Rad) to normalize chemiluminescent signals against total protein measured from the stain-free signal in the corresponding lane.

#### Lipid mass spectrometry

A total of 13,500 young-adult worms were hand picked for liquid chromatography- mass spectrometry (LC-MS) analyses. During the 1 h collection, worms were kept in 1 ml of M9 buffer. After gathering, the worms were gravity settled and washed three times with 1 ml of M9 buffer to remove bacteria. The buffer was aspirated almost entirely, and the worms were flash frozen in liquid nitrogen and shipped in dry ice to the core facility. Considering that the average residence time for a bacterium within the *C. elegans* intestine is <2 min (Avery and Shtonda, 2003; McGhee, 2007), we are confident that after the washes, these assays measure phosphoinositides that have been digested and incorporated into their respective tissues.

##### Phosphoinositide lipid class analysis

We collected 1000 worms (in triplicate) per strain and diet condition and shipped them to the Lipidomics Core Facility at Wayne State University (Detroit, MI). Lipid extraction was performed as described previously (Clark et al., 2011). Fifty nanograms of PI (16:0/16:0)-d7 was added to the worms before the single-phase extraction with a 2 CH_3_OH:1 CHCl_3_, 31 mm HCl solution. Samples were analyzed for PI, PIP, and PIP_2_ by direct infusion on a QTRAP 5500 (SCIEX) following published methods (Milne et al., 2005). Data were analyzed for phosphoinositide lipids using LipidView software (SCIEX).

##### Phosphoinositide lipid species analysis

We collected 500 worms per strain and diet condition and shipped them to the Mass Spectrometry Lipidomics Core Facility at the University of Colorado Anschutz Medical Campus (Aurora, CO). Lipids were extracted, and 1 µl of SPLASH LIPIDOMIX (Avanti Polar Lipids) was added to each sample as an internal standard. The data were normalized by integrating the area of each molecular species to that of the internal standard [15:0–18:1(d7) PI].

#### Calcium imaging

We measured Ca^2+^ influxes in live worms (Hilliard et al., 2005) using GCaMP to monitor *in vivo* activity of TRPV1-expressing ASH neurons upon exposure to capsaicin. Transgenic worms were glued (WormGlu, GluStich) on agar pads (2% agarose) and placed in a perfusion chamber under a constant flow of buffer solution (80 mm NaCl, 5 mm KCl, 5 mm MgCl_2_, 2 mm CaCl_2_, 25 mm sucrose, 10 mm HEPES, and 20 mm glucose, pH 7.3). This solution was used to perfuse capsaicin or SDS to the worms at the specified working concentrations. Solutions were applied via a gravity-fed perfusion system (AutoMate Scientific). TRPV1-mediated Ca^2+^ entry was elicited by perfusion of capsaicin through a needle placed near the tip of the head of the worm. The fluorescence intensity changes were acquired using an Olympus LUMPlan FL N 40×/0.080 W water-immersion objective and standard GFP filter, with an excitation wavelength of 488 nm and emission wavelength of 525 nm. Capsaicin-mediated fluorescence responses were normalized to SDS-maximal fluorescence intensity. Images were acquired and analyzed with CellSens Dimension (Olympus).

#### Cell culture and patch-clamp recordings

HEK293 cells (ATCC) were cultured in DMEM (Thermo Fisher Scientific), supplemented with 10% fetal bovine serum and 1% penicillin-streptomycin at 37°C and 5% CO_2_. Transfections were performed in six-well plates using Lipofectamine 2000 (Thermo Fisher Scientific), according to the manufacturer instructions, and recorded 18 h later.

##### Whole-cell recordings

HEK293 cells were cotransfected with 100–300 ng/ml rat TRPV1-pMO or 250–500 ng/ml Δ764-TRPV1-pMO and 50 ng/ml of GFP-pMO (a pcDNA3.1-based vector with the 5′ and 3′ untranslated regions of the beta-globin gene). The extracellular solution contained 140 mm NaCl, 6 mm KCl, 1 mm MgCl_2_, 10 mm glucose, 10 mm HEPES, pH 7.4, adjusted using NaOH. Recording electrode pipettes were made of borosilicate glass (Sutter Instrument) and fire polished to a resistance between 2.5 and 3.5 MΩ. Pipettes were filled with an intracellular solution containing 140 mm CsCl, 5 mm EGTA, 1 mm MgCl_2_, and 10 mm HEPES, pH 7.2, adjusted using CsOH. For capsaicin and pH recordings, solutions were applied via a gravity-fed perfusion system (ValveLink8.2, AutoMate Scientific). For capsaicin-response experiments, a capsaicin stock was diluted into the extracellular solution to reach the specified working concentration. Solutions were applied after entering into the whole-cell configuration. An initial low-agonist concentration solution (0.5 μm capsaicin or pH 7) was applied to the cell for a minimum of 25 s, then exchanged for a solution of a high concentration (10 μm capsaicin or pH 5.5) after five consecutive ramp sweeps did not show an increase in current magnitude. Cells were recorded under voltage-clamp conditions using an Axopatch 200A (Molecular Devices), with 1.5 s ramps from −80 to +80 mV, a sampling rate of 10 kHz, and analyzed offline using Clampfit version 10.4 (Molecular Devices).

##### Single-channel recordings

HEK293 cells were cotransfected with 5 ng/ml rat TRPV1-pMO or 50 ng/ml Δ764-TRPV1-pMO and 10 ng/ml GFP-pMO. Single-channel recordings were made in symmetrical conditions using a solution containing 130 mm NaCl, 3 mm HEPES, and 1 mm EDTA, pH 7.2, adjusted using NaOH. The agonist (0.5 μm capsaicin) and antagonist (5 μm capsazepine) perfused during the experiments were dissolved in the recording solution. Pipettes were made out of borosilicate glass (Sutter Instrument) and fire polished to a resistance between 4 and 5 MΩ. Single-channel currents were recorded in the inside-out configuration at a constant voltage (+60 mV), sampled at 20 kHz, and low-passed filtered at 2 kHz using a Multiclamp 700B amplifier and Clampex (Molecular Devices). Single-channel current amplitudes were determined by fitting a Gaussian function to all-points histograms, generated from 1 min recordings of three independent preparations. The nominal open probability (NPo) was determined from idealized 1 min traces beginning from agonist perfusion and defined as the sum of the total open time divided by the total length of the trace (1 min). The mean open time was defined as the average duration of all events in the open state for 1 min in the presence of 0.5 μm capsaicin. Idealization and analysis were done using ClampFit version 10.4 (Molecular Devices). Filter dead time was considered (∼90 µs) to discard events <100 µs.

#### Statistical analysis

Statistical analyses were performed using GraphPad InStat 3 software and Estimation Stats (Ho et al., 2019). Individual tests are described in each of the figure legends.

#### Data availability

Data supporting the findings of this manuscript are available from the corresponding authors upon reasonable request. The source data underlying figures are provided as a Source Data file, as follows: https://figshare.com/s/3d9c29a478b80a4925cb.

## Results

### Behavioral characterization of mammalian TRPV1 expressed in ASH neurons

**Figure 1.**
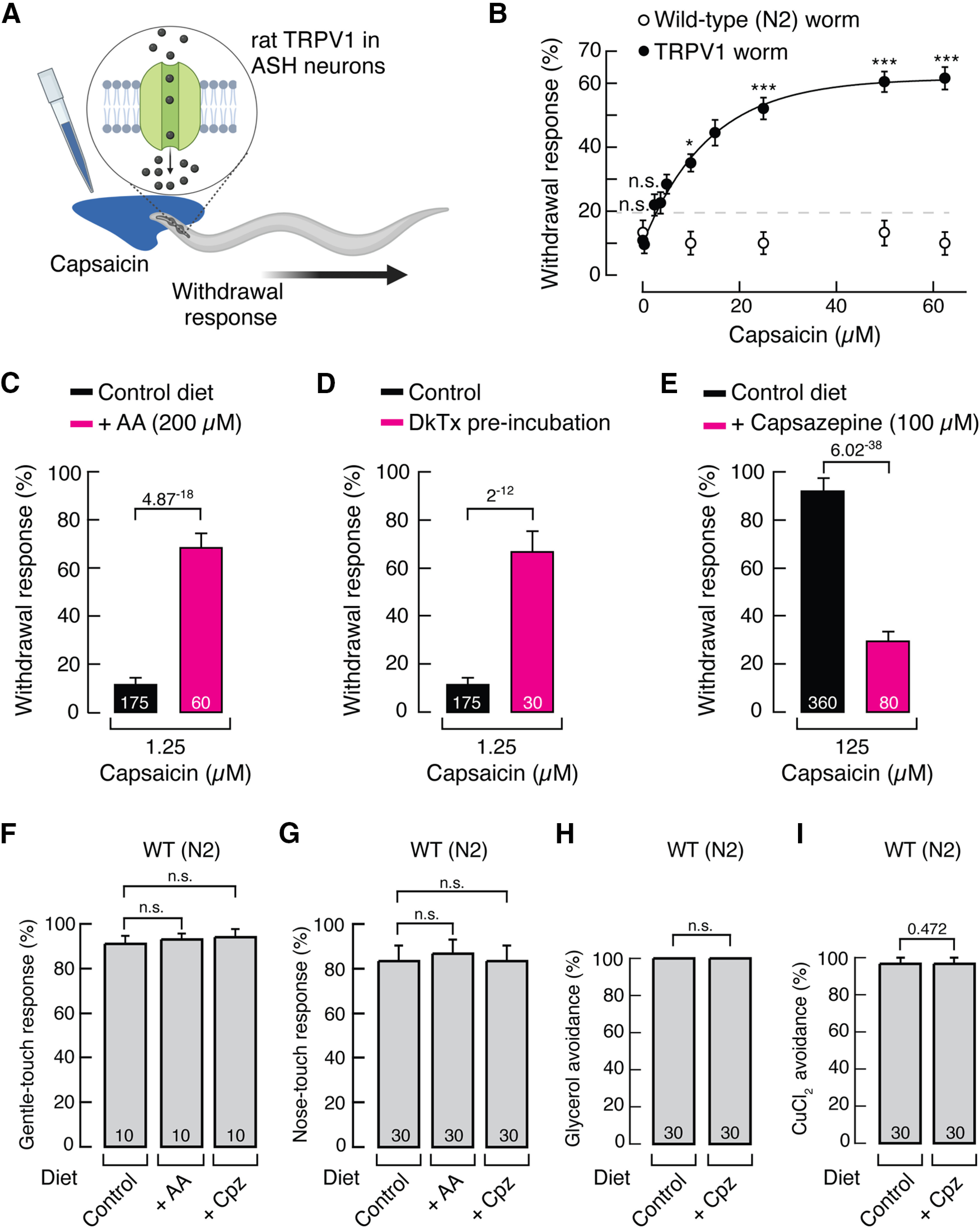
Behavioral characterization of rat TRPV1 worms. ***A***, Schematic representation of the aversive behavior (withdrawal responses) elicited after the addition of a capsaicin drop in front of a freely moving young adult worm. Created with BioRender.com. ***B***, Capsaicin dose–response profile for wild-type (WT or N2) and rat TRPV1 worms. Each circle represents the mean ± SEM, tested during at least three independent assay sessions. For N2, *n* ≥ 30 and for TRPV1 worm, *n* ≥ 60. Withdrawal responses >20% are considered positive withdrawal responses (dotted line). Kruskal–Wallis and Dunn's multiple-comparisons tests. Asterisks (∗∗∗*p* < 0.001 and ∗*p* < 0.05) indicate values significantly different from N2 worms. n.s., Not significant. ***C***, Withdrawal responses elicited by capsaicin (1.25 μm) of TRPV1 worms fed with control or AA-supplemented (200 μm) diets. ***D***, Withdrawal responses elicited by capsaicin (1.25 μm) of TRPV1 worms with or without incubation with DkTx (5 μm) for 15 min. ***E***, Withdrawal responses elicited by capsaicin (125 μm) of TRPV1 worms fed with control and capsazepine-supplemented (100 μm) diets. ***F***, Withdrawal responses elicited by 10-trial gentle body touch assay of WT (N2), after animals were fed with control, AA- or capsazepine-supplemented diets. ***G***, Withdrawal responses elicited by nose touch of WT (N2), after animals were fed with control, AA-supplemented, or capsazepine-supplemented diets. ***H***, Withdrawal responses elicited by high osmolarity (glycerol 1 m) of WT (N2), after animals were fed with control or capsazepine-supplemented diets. ***I***, Withdrawal responses elicited by CuCl_2_ (1 mm) of WT (N2), after animals were fed with control or capsazepine-supplemented diets. ***C–I***, Bars are the mean ± SEM; the number of worms tested during at least three independent assays are indicated inside the bars. ***C–E***, ***H***, ***I***, Mann–Whitney rank test for two independent groups. ***F***, ***G***, Kruskal–Wallis and Dunn's multiple-comparisons tests. *p* Values are denoted above the bars. n.s. indicates values are not significantly different.

We first characterized the behavioral response of an integrated transgenic *C. elegans* strain expressing rat TRPV1 (not encoded in the wild-type worm genome) in a set of sensory neurons termed ASH (Tobin et al., 2002). Behavioral trials were performed by placing drops (Hart, 2006) of different capsaicin concentrations in front of moving worms (Fig. 1*A*). Those trials that elicited reversals of motion (i.e., aversive behavior) were scored as withdrawal responses. At least 30 young adult hermaphrodite worms were tested in the trial each day (blind to genotype and treatment), and the results were compared across three trials. Capsaicin evoked aversive behavior in TRPV1 worms (EC_50_ = 11.66 ± 3.48 μm; mean ± SD), but not in wild-type (N2) worms (Fig. 1*B*; Kahn-Kirby et al., 2004). The requirement of high concentrations (≥10 μm) of capsaicin to evoke the withdrawal behavior of the worms was initially observed by Kahn-Kirby et al. (2004) when evaluating transgenic worms expressing rat TRPV1. Likewise, our effective capsaicin concentrations are similar to the ones obtained by Bargmann's group and lower than the concentrations needed to generate nociceptive responses in mice behavioral experiments (320–1200 μm; Caires et al., 2015; Nersesyan et al., 2017). We considered withdrawal responses >20% as evidence of evoked escape behavior; values below this threshold were considered random responses (Fig. 1*B*, dotted line).

To determine whether the capsaicin-aversive behavior of the worms could be positively or negatively regulated via TRPV1, we used molecules known to modulate TRPV1 function in mammalian cells. To this end, we supplemented the NGM with AA, a fatty acid known to enhance TRPV1 responses to other stimuli (Cao et al., 2013b) and that can be enriched in worms through diet supplementation (Kahn-Kirby et al., 2004). We found that TRPV1 worms displayed higher withdrawal responses to a lower concentration of capsaicin (1.25 μm) when AA is enriched in the NGM, compared with control diet worms (Fig. 1*C*). Likewise, capsaicin elicited robust withdrawal responses in TRPV1 worms that were preincubated with a bivalent tarantula toxin, when compared with control animals (DkTx; Fig. 1*D*). This result was expected since DkTx binds to and stabilizes TRPV1 in an open state by interacting with the pore-forming region of the channel (Bohlen et al., 2010; Cao et al., 2013a; Bae et al., 2016; Gao et al., 2016; Geron et al., 2018). We further tested the ability of capsazepine to inhibit TRPV1-mediated behavior and found a significant decrease in the withdrawal responses of worms to 125 μm capsaicin (Fig. 1*E*).

Importantly, N2 worms (strain that does not express TRPV1) grown on NGM supplemented with AA or capsazepine displayed normal responses to gentle body and nose touch, high osmolarity (i.e., glycerol), or CuCl_2_ (Fig. 1*F–I*). In addition, Kahn-Kirby et al. (2004) demonstrated that AA supplementation does not modify the N2 responses of worms to other sensory modalities, such as diacetyl and benzaldehyde chemotaxis or nose touch and high osmolarity avoidance. Moreover, we have previously shown that DkTx does not elicit withdrawal responses in N2 worms (Geron et al., 2018). Our behavioral results demonstrate that rat TRPV1 expressed in the neurons of worms elicits a specific and robust aversive reaction when challenged by capsaicin, and that the responses can be either positively or negatively modulated. This assay allows us to establish a suitable model for evaluating TRPV1 activity *in vivo*.

### Deficiency of inositol monophosphatase activity enhances TRPV1 function *in vivo*

**Figure 2.**
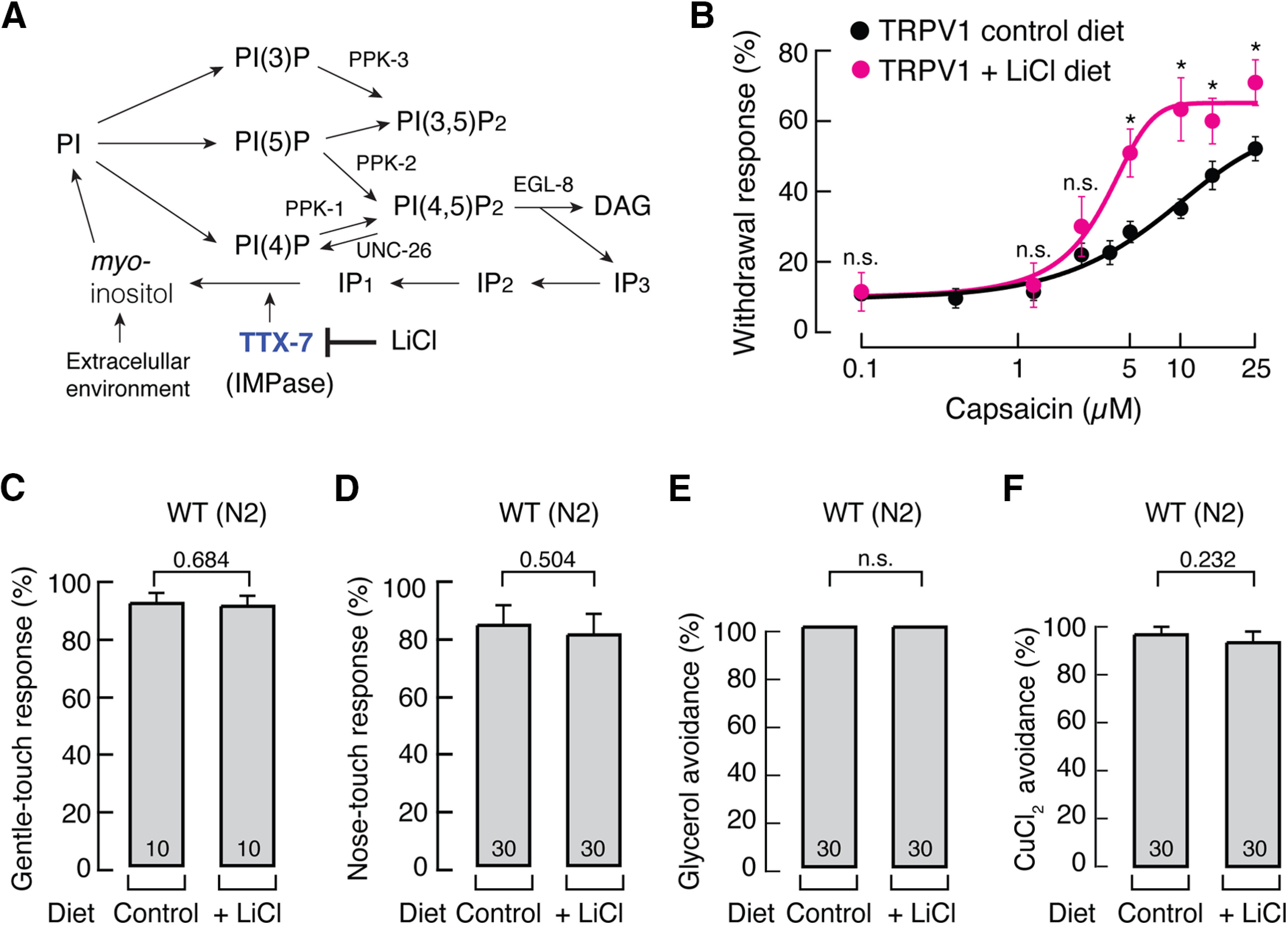
Chemical inhibition of inositol monophosphatase enhances TRPV1 function *in vivo*. ***A***, *C. elegans* phosphoinositide cascade, adapted from Kimata et al. (2012). TTX-7, *myo*-inositol monophosphatase; PPK, phosphatidylinositol phosphate kinase; UNC-26, synaptojanin; EGL-8, phospholipase C. Blue, Lipid-deficient mutant strain. ***B***, Capsaicin dose–response profile of TRPV1 worms fed with control or LiCl-supplemented (7.5 mm) diet. Each circle represents the mean ± SEM, tested during at least three independent assay sessions. Lines are Boltzmann functions fit to the data. For TRPV1 worms, *n* ≥ 60, and for TRPV1 worms supplemented with LiCl, *n* ≥ 30. Kruskal–Wallis and Dunn's multiple-comparisons tests. Asterisks indicate values significantly different from control (∗*p* < 0.05) and n.s. indicates values not significantly different from the control diet. ***C***, Withdrawal responses of WT (N2) worms elicited by gentle body touch after animals were fed with control or LiCl-supplemented diets. ***D***, Withdrawal responses of WT (N2) worms elicited by nose touch after animals were fed with control or LiCl-supplemented diets. ***E***, Withdrawal responses of WT (N2) worms elicited by high osmolarity (glycerol 1 m) after animals were fed with control or LiCl-supplemented diets. ***F***, Withdrawal responses of WT (N2) worms elicited by CuCl_2_ (1 mm) after animals were fed with control or LiCl-supplemented diets. ***C–F***, Bars are the mean ± SEM. The number of worms tested are indicated inside the bars. Mann–Whitney rank test for two independent groups. *p* Values are denoted above the bars. n.s. indicates values are not significantly different.

Cao et al. (2013b) demonstrated that a broad range of phosphoinositide lipids present in biological membranes negatively regulate TRPV1 activity in TRPV1-containing liposomes. *C. elegans* synthesizes this class of phosphoinositide lipids and, in particular, generates PI from environmental *myo*-inositol (i.e., coming from the NGM) and by recycling from other inositol lipids (Kimata et al., 2012). The latter requires a conserved IMPase (TTX-7) to synthesize *myo*-inositol by dephosphorylating inositol monophosphate (Fig. 2*A*; Kimata et al., 2012). It is expected that a loss of function of the TTX-7 enzyme produces worms with reduced levels of phosphoinositide lipids (Tanizawa et al., 2006; Kimata et al., 2012). To determine whether phosphoinositide lipids are positive or negative regulators of TRPV1 activity *in vivo*, we initially tested capsaicin-elicited responses in worms whose diet was supplemented by LiCl, a TTX-7 enzyme inhibitor (Kimata et al., 2012; Fig. 2*A*). We found that, in this condition, worms displayed an enhanced response to capsaicin, as shown by a leftward shift in the dose–response curve, indicating an increase in sensitivity to capsaicin (EC_50_ for control 11.66 ± 3.48 μm vs LiCl 3.42 ± 0.81 μm, mean ± SD; Fig. 2*B*). Notably, N2 worms grown on food supplemented with LiCl displayed normal responses to gentle body and nose touch, high osmolarity (i.e., glycerol), or CuCl_2_ (Fig. 2*C–F*).

**Figure 3.**
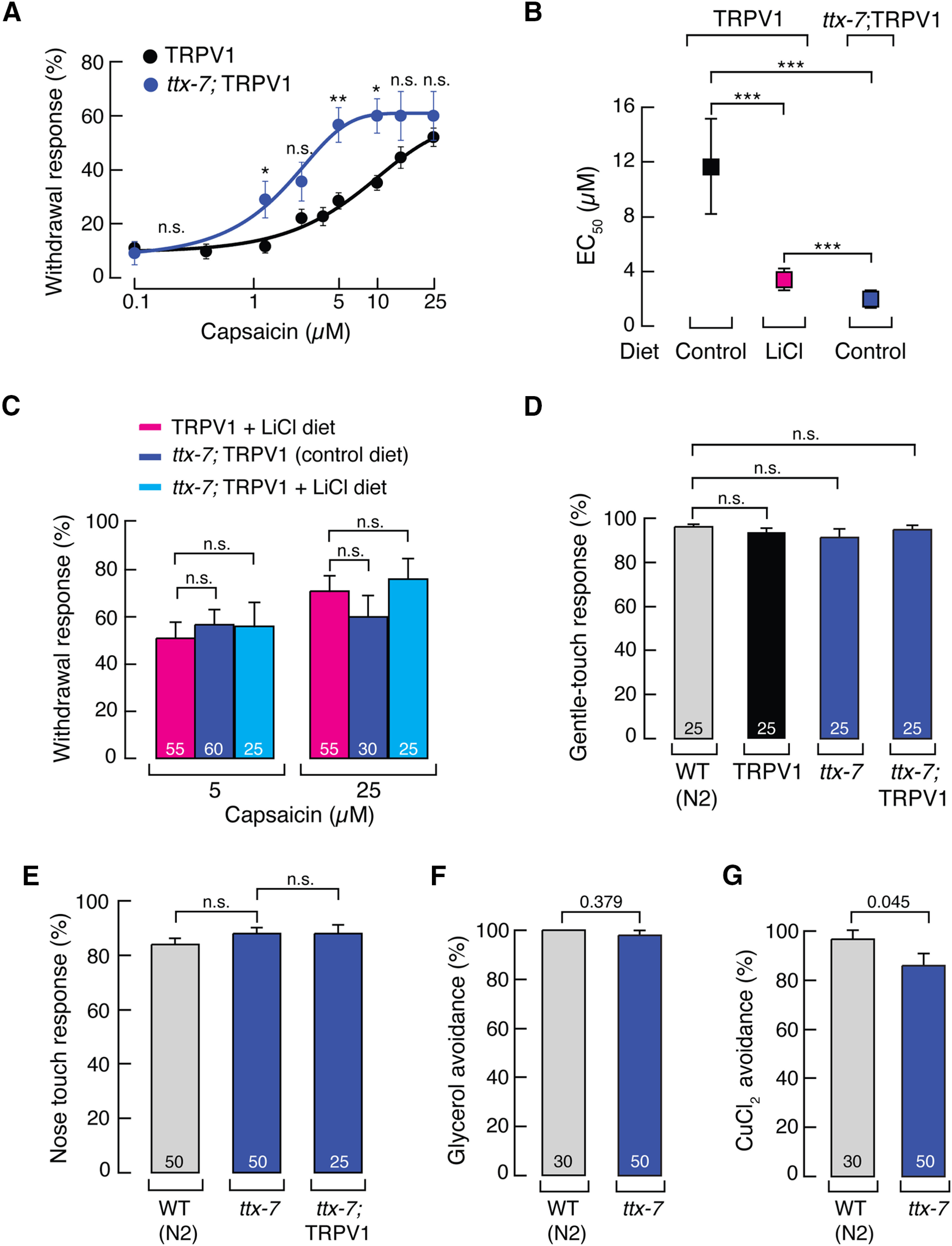
Lack of inositol monophosphatase activity enhances TRPV1 function *in vivo*. ***A***, Capsaicin dose–response profile of TRPV1 and *ttx-7*; TRPV1 worms. Each circle represents the mean ± SEM, tested during at least three independent assay sessions. Lines are Boltzmann functions fit to the data. For TRPV1 worms, *n* ≥ 60 and for *ttx-7*; TRPV1 worms, *n* ≥ 30. Kruskal–Wallis and Dunn's multiple-comparisons tests. Asterisks indicate values significantly different from control (∗∗*p* < 0.01 and ∗*p* < 0.05), and n.s. indicates values not significantly different from the TRPV1 worms. ***B***, EC_50_ for experiments shown on Figures 2*B* and 3*A*. Each square represents the EC_50_ ± SD from the Boltzmann function fit to the data. One-way ANOVA and Tukey–Kramer multiple-comparisons test. Asterisks indicate values significantly different from control (∗∗∗*p* < 0.001). ***C***, Withdrawal responses of TRPV1 and *ttx-7*; TRPV1 worms elicited by capsaicin (5 or 25 μm), after animals were fed with control or LiCl-supplemented diets. ***D***, Withdrawal responses elicited by gentle body touch of WT (N2), TRPV1, *ttx-7*, and *ttx-7*; TRPV1 worms. ***E***, Withdrawal responses of WT (N2), *ttx-7*, and *ttx-7*; TRPV1 worms elicited by nose touch. ***F***, Withdrawal responses of WT (N2) and *ttx-7* worms elicited by high osmolarity (glycerol 1 m). ***G***, Withdrawal responses of WT (N2) and *ttx-7* worms elicited by CuCl_2_ (1 mm). ***C–G***, Bars are the mean ± SEM. The number of worms tested are indicated inside the bars. ***C–E***, Kruskal–Wallis and Dunn's multiple-comparisons tests. ***F***, ***G***, Mann–Whitney rank test for two independent groups. *p* Values are denoted above the bars. n.s. indicates values are not significantly different.

We next crossed TRPV1 worms with a strain lacking the function of the TTX-7 enzyme (Fig. 2*A*) and assessed the behavioral responses on this genetic background (*ttx-7*; TRPV1). Remarkably, we found that these animals exhibited an enhanced response to capsaicin, with a decrease in the EC_50_ from 11.66 ± 3.48 to 1.98 ± 0.65 μm (mean ± SD; control and *ttx-7*, respectively; Fig. 3*A*,*B*). Of note, we did not observe significant differences in capsaicin-mediated withdrawal behavior between *ttx-7*; TRPV1 worms grown with or without LiCl (Fig. 3*C*). This result suggests that the effect of LiCl on TRPV1-mediated behavior is mostly through inhibition of the inositol monophosphate enzyme pathway, rather than a nonspecific pathway. In worms, gentle body touch is mediated by the mechanoelectrical transduction channel complex expressed in touch receptor neurons (Goodman, 2006), while nose touch and glycerol avoidance response are principally mediated by DEG-1 and OSM-9 in ASH neurons, respectively (Goodman, 2006; Geffeney et al., 2011), and soluble-repellent (CuCl_2_) avoidance is primarily mediated by the G-protein α-subunit (worm GPA-3) expressed in ASH neurons (Hilliard et al., 2004; Ezcurra et al., 2011). These sensory modalities were not affected by genetically impairing TTX-7 function (Fig. 3*D–G*). Furthermore, Tanizawa et al. (2006) demonstrated that *ttx-7* worm chemotaxis does not significantly differ from N2 worms. Together, these results support that TRPV1 displays enhanced activity in worms whose TTX-7 enzyme function was chemically or genetically impaired.

### Phosphoinositide lipid supplementation decreases TRPV1 function *in vivo*

**Figure 4.**
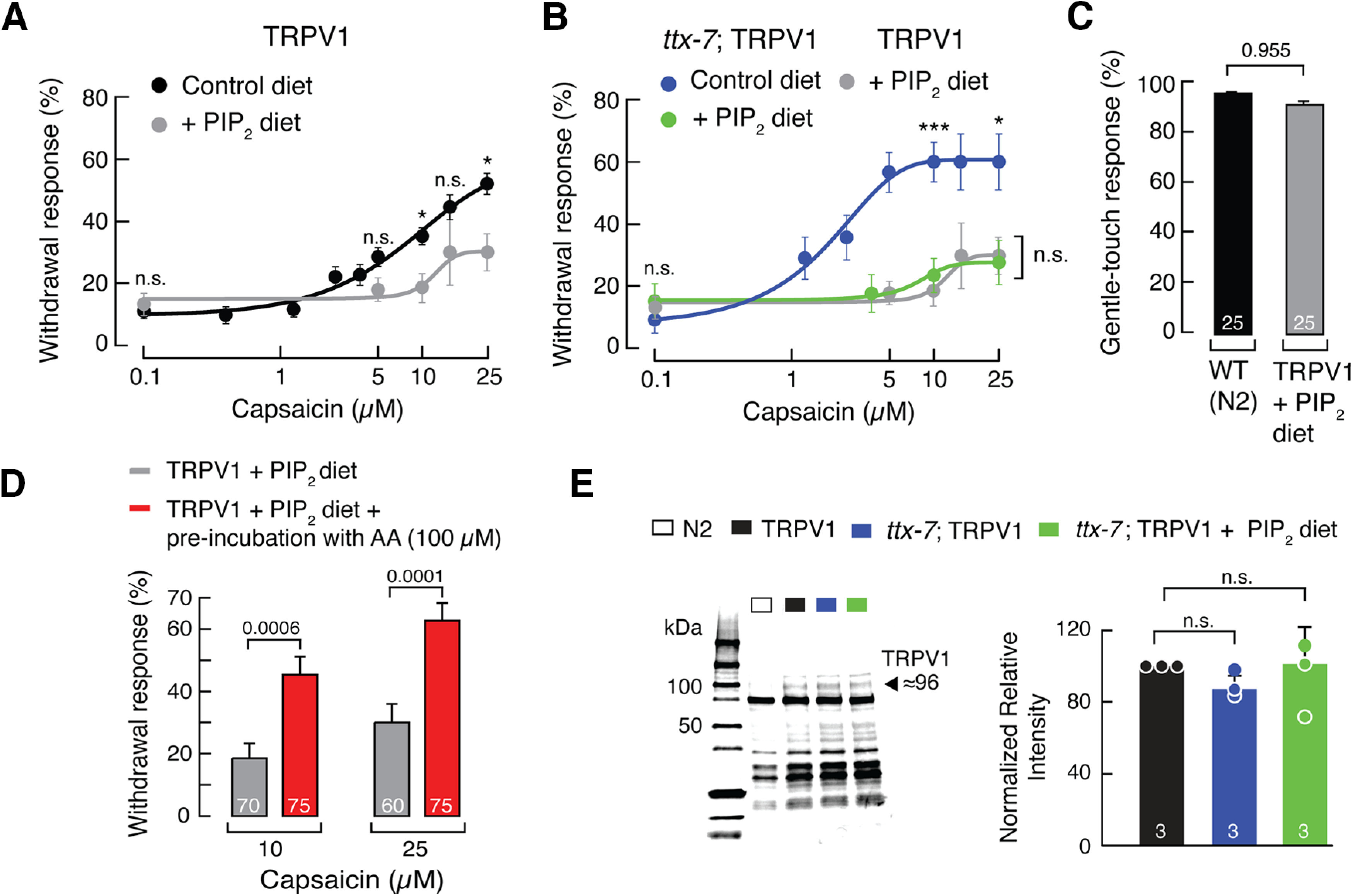
Phosphoinositide lipid supplementation decreases TRPV1 function *in vivo*. ***A***, Capsaicin dose–response profile of TRPV1-expressing worms fed with control or PIP_2_-supplemented (200 μm) diets. Each circle represents the mean ± SEM, tested during at least three independent assay sessions. Lines are Boltzmann functions fit to the data. For control diet, *n* ≥ 60 and for PIP_2_-supplemented diet, *n* ≥ 20. Kruskal–Wallis and Dunn's multiple-comparisons tests. Asterisks indicate values significantly different from control diet (∗*p* < 0.05). n.s. indicates values are not significantly different. ***B***, Capsaicin dose–response profiles of *ttx-7*; TRPV1 worms fed with control or PIP_2_-supplemented (200 μm) diet. TRPV1 worms fed with PIP_2_-supplemented (200 μm) diet (gray curve) are included for reference. Each circle represents the mean ± SEM, tested during at least three independent assay sessions. Lines are Boltzmann functions fit to the data. For control diet, *n* ≥ 30; for PIP_2_-supplemented diet, *n* ≥40. Kruskal–Wallis and Dunn's multiple-comparisons tests. Asterisks indicate values significantly different from control (∗∗∗*p* < 0.001 and ∗*p* < 0.05). n.s. indicates values are not significantly different. ***C***, Withdrawal responses elicited by gentle body touch of WT (N2) and TRPV1 worms fed with PIP_2_-supplemented (200 μm) diet. Bars are the mean ± SEM; the number of worms tested during at least three independent assays are indicated inside the bars. Mann–Whitney rank test for two independent groups. The *p* value is denoted above the bars. ***D***, Arachidonic acid potentiates the response of TRPV1 worms supplemented with PIP_2_ diet. Withdrawal responses elicited by capsaicin (10 and 25 μm), after TRPV1 worms were fed with PIP_2_-supplemented (200 μm) diet and preincubated for 5 min with AA (100 μm). Bars are the mean ± SEM; the number of worms tested are indicated inside the bars. Mann–Whitney rank test for independent groups. *p* Values are denoted above the bars. ***E***, Left, western blot with anti-TRPV1 antibody of the membrane fractions of N2, TRPV1, *ttx-7*; TRPV1, and *ttx-7*; TRPV1 + PIP_2_ worms. The TRPV1 predicted molecular weight is 94.95 kDa. Right, Relative intensities calculated from total protein detection of chemically labeled proteins (Stain-Free System Bio-Rad) of the membrane fractions of N2, TRPV1, *ttx-7*; TRPV1, and *ttx-7*; TRPV1 + PIP_2_ worms normalized against TRPV1 worms. Bars are the mean ± SD; the number of western blots is indicated inside the bars. Kruskal–Wallis and Dunn's multiple-comparisons tests. n.s. indicates values are not significantly different.

If TRPV1 function is enhanced in worms deficient in the activity of the TTX-7 enzyme, we would expect that increasing the phosphoinositide lipid content should decrease channel function. To this end, we added a natural lipid PIP_2_ mixture (porcine brain PIP_2_) highly enriched in 18:0 and 20:4 acyl chains as a source of phosphoinositide lipids to the diet of the worms. We supplemented phosphoinositide lipids via the diet in the same way that we, and others, have previously supplemented fatty acids (Watts and Browse, 2002; Kahn-Kirby et al., 2004; Vásquez et al., 2014; Caires et al., 2017), cholesterol and cholesterol derivatives (Huber et al., 2006), RNAi (Kamath et al., 2001), and all-*trans*-retinol (Nagel et al., 2005). We found that supplementing the diet of TRPV1 worms with PIP_2_ decreases capsaicin-aversive behavior (Fig. 4*A*). Remarkably, *ttx-7*; TRPV1 worms supplemented with the PIP_2_ diet displayed a decreased aversive response, evident by the robust rightward shift in the dose–response curve, when compared with control diet (Fig. 4*B*). This result was expected since even in the absence of TTX-7 enzyme activity, other enzymes in the phosphoinositide cascade (e.g., UNC-26, EGL-8; Fig. 2*A*) can restore worm phosphoinositide lipid content. When comparing TRPV1 versus *ttx-7*; TRPV1 worms supplemented with the PIP_2_ diet, we did not observe significant differences in their withdrawal responses (Fig. 4*B*). Hence, phosphoinositide diet supplementation is sufficient to prevent the *ttx-7*; TRPV1-worms' enhanced aversive response. To determine whether the decrease in withdrawal responses in phosphoinositide lipid-supplemented worms originated from an effect in TRPV1 function rather than a global behavioral decay, we challenged the touch receptor neurons of these worms with mechanical stimulation. TRPV1 worms supplemented with PIP_2_ displayed wild-type-like aversive responses to gentle body touch (Fig. 4*C*).

We next tested the ability of AA to increase withdrawal responses in TRPV1 worms supplemented with PIP_2_, since TRPV1 function can be potentiated with this fatty acid (Fig. 1*C*). Indeed, we found that acute incubation with AA before capsaicin exposure increases TRPV1-mediated behavioral response (Fig. 4*D*), suggesting that TRPV1 channels can still be potentiated by AA when the total phosphoinositide lipid content of the worm is high. Furthermore, we observed no obvious differences in TRPV1 membrane expression levels among TRPV1, *ttx-7*; TRPV1, and *ttx-7*; TRPV1 + PIP_2_ worm strains after western blot analyses (Fig. 4*E*), supporting the idea that behavioral responses are not because of differences in channel expression.

### Deficiency of TTX-7 enzyme activity decreases phosphoinositide lipid content

**Figure 5.**
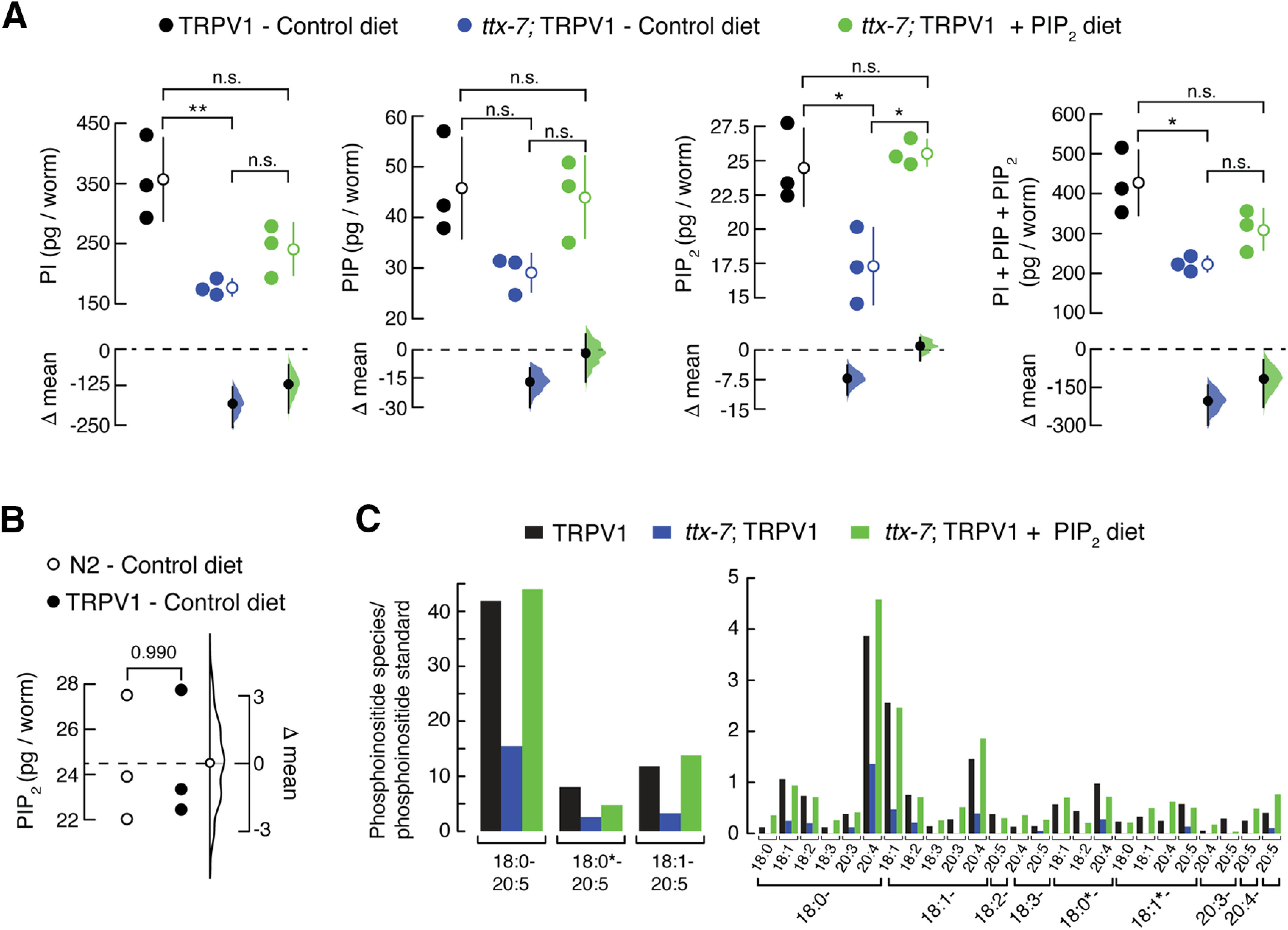
Phosphoinositide lipid content determination of TRPV1 worms. ***A***, Cumming estimation plots showing the mean differences in PI, PIP, and PIP_2_ content between TRPV1 worms fed with control diet and *ttx-7*; TRPV1 worms fed with control or PIP_2_-supplemented (200 μm) diet. The raw data are plotted on the upper axes (*n* = 3; 1000 worms per replicate); each mean difference is plotted on the lower axes as a bootstrap sampling distribution. The mean differences are depicted as dots; 95% confidence intervals are indicated by the ends of the vertical error bars. One-way ANOVA and Tukey–Kramer multiple-comparisons test. Asterisks indicate values significantly different from control (∗∗*p* < 0.01 and ∗*p* < 0.05); n.s. indicates values not significantly different from the control. ***B***, Gardner–Altman estimation plot showing the mean difference in PIP_2_ content between N2 and TRPV1 worms fed with control diet. The raw data are plotted on the left axis. The mean difference, on the right, is depicted as a dot; the 95% confidence interval is indicated by the ends of the vertical error bars. Two-tailed unpaired *t* test. *p* Value is denoted above the plot. ***C***, Quantification of esterified long fatty-acyl chains in phosphoinositide lipids of TRPV1 and *ttx-7*; TRPV1 worms or *ttx-7*; TRPV1 worms fed with PIP_2_-supplemented (200 μm) diet, as determined by LC-MS. Abundant and less abundant lipid species are plotted separately on the left and right, respectively. The nomenclature 18:0–20:5 indicates fatty acyl moieties as the number of carbons:acyl double bonds for each species. Molecular species designated with a * could contain either an ether or an odd-carbon acyl group; for instance, 18:0*−20:5 also could be 17:0-20:5. Five hundred young adult worms were used for the quantification.

It is challenging to differentiate between the polar head groups of phosphoinositide lipid classes because of rapid hydrolysis and detection sensitivity. Although mass spectrometry profiling of phosphoinositide lipids is hampered by their high polarity and low cellular concentrations (Kielkowska et al., 2014; Traynor-Kaplan et al., 2017), we were able to quantify the phosphoinositide lipid classes PI, PIP, and PIP_2_ of the TRPV1 worms. For LC-MS analyses, we manually collected a total of 9000 worms (1000 worms per condition, in triplicate), which is sufficient to detect differences in the phosphoinositide lipid classes. Quantification of PI, PIP, and PIP_2_ content features a significant decrease in which *ttx-7*; TRPV1 worms (blue circles) contain fewer of these lipid classes when compared with TRPV1 worms (Fig. 5*A*). On the other hand, when supplementing their diet with porcine brain PIP_2_, we found an increasing trend for PI, PIP, and PIP_2_ (Fig. 5*A*; green circles). Notably, when supplementing the diet with PIP_2_, we observed a robust increase in this lipid when compared with PI and PIP (Fig. 5*A*). Parenthetically, we determined the PIP_2_ content of N2 worms (3000 worms total; 1000 worms per sample) and found similar amounts of PIP_2_ when compared with TRPV1 worms (both fed with the control diet; Fig. 5*B*).

We also quantified the phosphoinositide lipid species by analyzing the spectrum of esterified fatty-acyl chains contained in TRPV1 worms (i.e., phosphoinositide lipids contain different combinations of fatty acyl-chains, with C18 and C20 being the most abundant ones in biological membranes). We collected a total of 500 worms for each condition (one replicate) and determined that *ttx-7*; TRPV1 worms contain ∼65% fewer phosphoinositide species than control worms (Fig. 5*C*). The remaining ∼35% of phosphoinositide lipids on the *ttx-7* background likely comes from the environmental *myo*-inositol, which is sufficient for maintaining normal *C. elegans* growth and physiology. Moreover, *ttx-7*; TRPV1 worms supplemented with PIP_2_ contained similar amounts of esterified fatty-acyl chains in phosphoinositide species when compared with TRPV1 worms (Fig. 5*C*). This result highlights that the supplementation protocol in *ttx-7*; TRPV1 worms increased the amount of total phosphoinositide species to nearly wild-type levels. Importantly, worms are able to properly metabolize the phosphoinositide lipids supplemented in the diet, since in the lipid species quantification analyses, we detected high levels of 18:0–20:5 compared with the 18:0–20:4 provided in the diet (Fig. 5*C*). The main reason for this change is the ability of the worms to add an unsaturation to the *ω*−6 20:4 fatty acid to convert it into *ω*−3 20:5 (via the *ω*−3 fatty acid desaturase FAT-1). Together, results from behavioral and LC-MS analyses support the idea that TRPV1 displays enhanced activity in worms where the content of phosphoinositide lipids is reduced.

### Phosphoinositide lipid supplementation decreases Ca^2+^ transients in TRPV1-expressing ASH neurons

**Figure 6.**
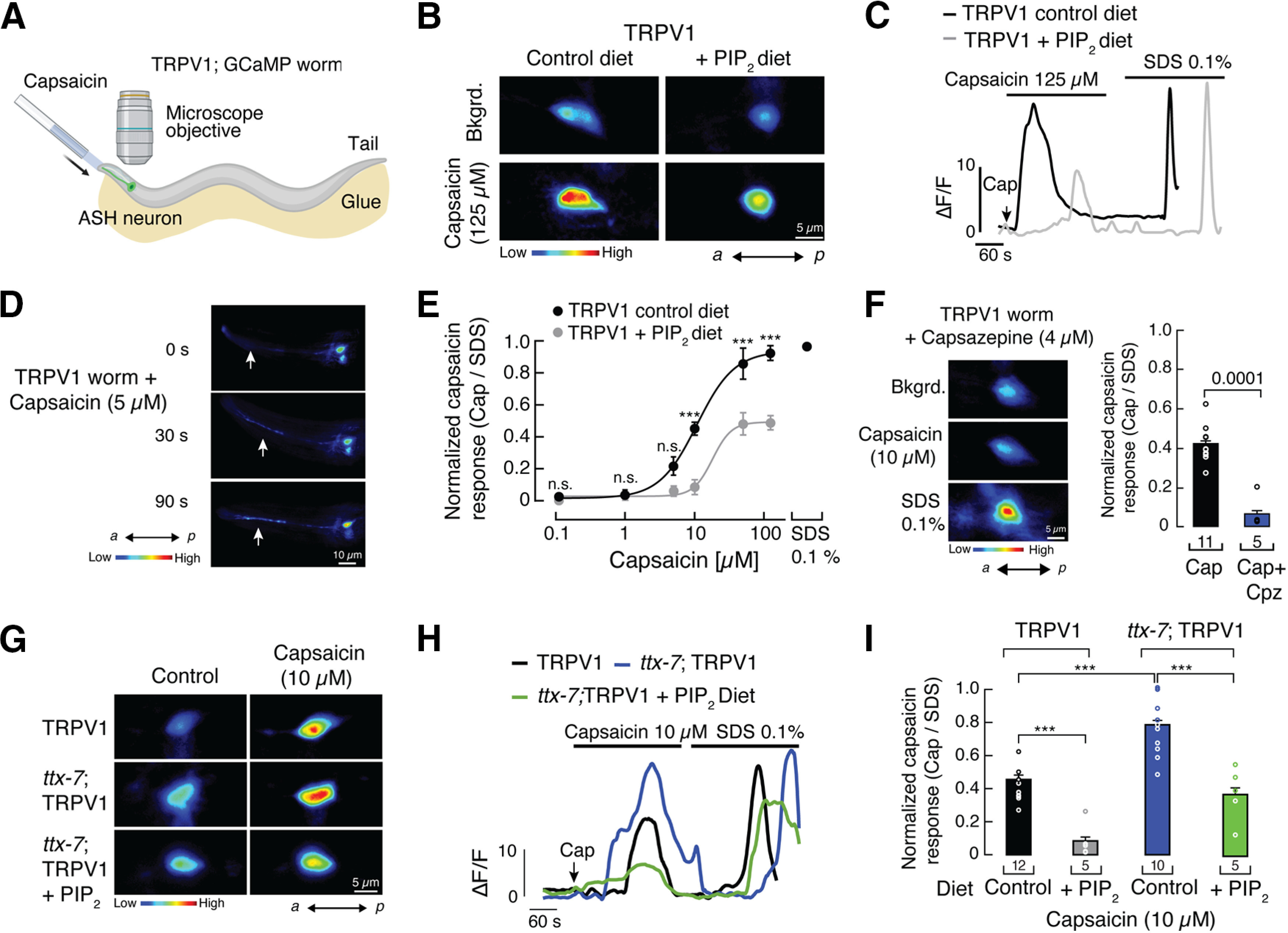
Functional characterization of TRPV1 and *ttx-7*; TRPV1-GCaMP worms. ***A***, Schematic representation of the setup for *in vivo* Ca^2+^ imaging of ASH neurons. Created with BioRender.com. ***B***, Micrographs of ASH neurons challenged with capsaicin (125 μm) in worms fed with control or PIP_2_-supplemented (200 μm) diet. Bkgrd, Background. Color bar indicates the relative change in fluorescence intensity, with blue and red denoting the lowest and highest cytoplasmic Ca^2+^, respectively (adjustment display set to 10–75). *a*, Anterior; *p*, posterior. ***C***, Representative traces corresponding to fluorescence intensity changes (Δ*F*/*F*) of individual cells shown in ***B***. ***D***, Representative time-lapse micrographs of ASH neurons challenged with capsaicin (5 μm) in TRPV1-GCaMP worms. Color bar indicates the relative change in fluorescence intensity, with blue and red denoting the lowest and highest cytoplasmic Ca^2+^, respectively (adjustment display set to 8–65). *a*, Anterior; *p*, posterior. ***E***, Capsaicin dose–response profile for ASH neurons challenged with capsaicin in worms fed with control or PIP_2_-supplemented diet. The capsaicin response was normalized with the response elicited by 0.1% SDS. Lines are Boltzmann functions fit to the data. For control diet, *n* ≥ 3; for PIP_2_ diet, *n* ≥ 4. One-way ANOVA and Bonferroni *post hoc* tests. Asterisks indicate values significantly different from control (∗∗∗*p* < 0.001); n.s. indicates values are not significantly different. ***F***, Left, Representative micrographs of ASH neurons challenged with capsaicin (10 μm) and SDS (0.1%) in TRPV1-GCaMP worms fed with capsazepine-supplemented (4 μm) diet (adjustment display set to 10–75). Color bar indicates the relative change in fluorescence intensity, with blue and red denoting the lowest and highest cytoplasmic Ca^2+^, respectively. *a*, Anterior; *p*, posterior. Right, Normalized response for ASH neurons challenged with capsaicin (10 μm) in TRPV1-GCaMP worms fed with capsazepine-supplemented (4 μm) diet. The capsaicin response was normalized with the response elicited by 0.1% SDS. Bars are the mean ± SEM; *n* is denoted above the *x*-axis. Two-tailed unpaired *t* test for independent groups. *p* Value is denoted above the bar. ***G***, Representative micrographs of ASH neurons challenged with capsaicin (10 μm) in TRPV1 and *ttx-7*; TRPV1-GCaMP worms fed with control or PIP_2_-supplemented (200 μm) diets. Color bar indicates the relative change in fluorescence intensity, with blue and red denoting the lowest and highest cytoplasmic Ca^2+^, respectively (adjustment display set to 10–75). *a*, Anterior; *p*, posterior. ***H***, Representative traces corresponding to fluorescence intensity changes (Δ*F*/*F*) of individual cells shown in ***G***. ***I***, Normalized response for ASH neurons challenged with capsaicin (10 μm) in TRPV1 and *ttx-7*; TRPV1 worms fed with control or PIP_2_-supplemented (200 μm) diets. The capsaicin response was normalized with the response elicited by 0.1% SDS. Bars are the mean ± SEM; *n* is denoted above the *x*-axis. One-way ANOVA and Tukey–Kramer multiple-comparisons test. Asterisks indicate values significantly different (∗∗∗*p* < 0.001).

To assess the functionality of rat TRPV1 in ASH neurons, we imaged live worms using a GCaMP (Fig. 6*A*; Hilliard et al., 2005). We measured the fluorescence intensity changes in ASH neuron somas after exposure to capsaicin as a readout for TRPV1-mediated Ca^2+^ influx. As expected, capsaicin elicits Ca^2+^ transients in neurons of TRPV1 worms (Fig. 6*B*, left panels, *C*), which can be observed progressively increasing from the neurites (anterior) to the soma (posterior) even at low capsaicin concentration (5 μm; Fig. 6*D*). TRPV1 worms fed with a PIP_2_-enriched diet displayed decreased Ca^2+^ transients (Fig. 6*B*, right panels, *C*) and a rightward shift in the capsaicin dose–response curve when compared with worms fed with the control diet [EC_50_ range, 10.80 ± 1.58 to 19.11 ± 8.32 μm (mean ± SD), control and PIP_2_, respectively; Fig. 6*E*]. The decrease in Ca^2+^ influx found in worms fed with PIP_2_ (likely diminishing neuron depolarization) correlates with the reduction of withdrawal responses observed in worms whose diet was supplemented with PIP_2_ (Fig. 4*A*,*B*). Notably, the Ca^2+^ transients in neurons of TRPV1 worms can be inhibited by adding capsazepine to the diet (Fig. 6*F*), supporting the idea that TRPV1 drives mainly the Ca^2+^ transients when challenged with capsaicin.

If the Ca^2+^ transients in neurons correlate with aversive behavior, we would expect to observe larger Ca^2+^ transients in worms with reduced levels of phosphoinositide lipids (*ttx-7*; TRPV1). Indeed, when challenged with capsaicin, we measured large Ca^2+^ transients in worms lacking the enzymatic activity of TTX-7, when compared with control worms (Fig. 6*G*, top and middle panels, *H*, *I*). As observed in the behavioral assay (decreased aversive response), *ttx-7*; TRPV1 worms fed with a phosphoinositide lipid source display smaller capsaicin-mediated Ca^2+^ transients than worms with the control diet (Fig. 6*G*, bottom panels, *H*, *I*). Importantly, the Ca^2+^-imaging results highlighted that TRPV1 modulation by phosphoinositide lipids is not because of nonspecific effects elsewhere in the circuit of worms. Together, our Ca^2+^-imaging experiments in live worm ASH neurons (independent of the behavioral approach) demonstrate that TRPV1 function is enhanced with reduced levels of phosphoinositide lipids and decreased with higher levels.

### TRPV1 C-terminal domain is required for the phosphoinositide lipid-mediated inhibition

**Figure 7.**
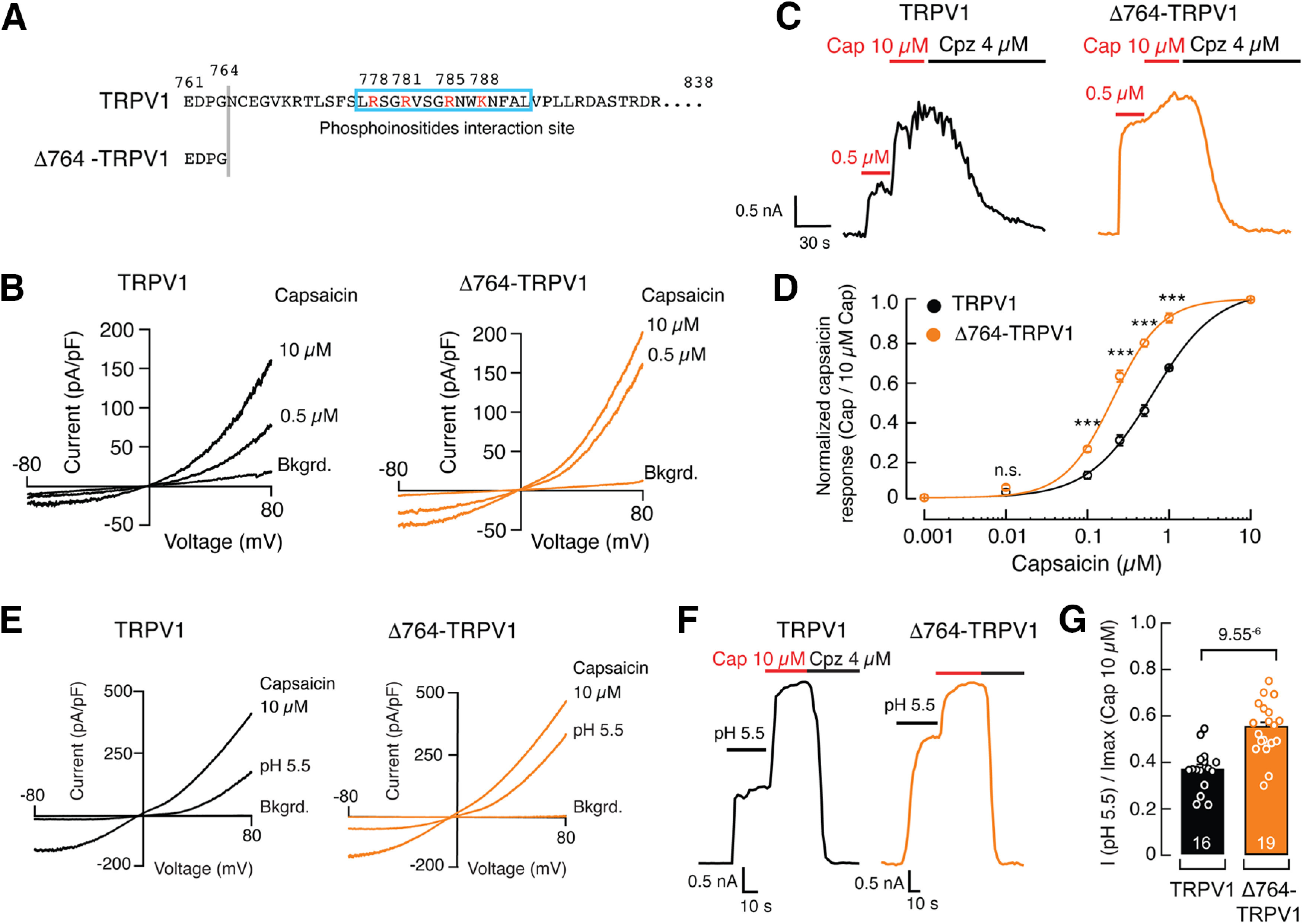
Capsaicin and pH responses of the truncated TRPV1 C-terminal domain construct Δ764-TRPV1. ***A***, Amino acid residue sequence highlighting the missing distal C-terminal domain including the phosphoinositide interaction site (cyan box) in the Δ764-TRPV1 construct. Constructs were transfected and measured in HEK293 cells. ***B***, Representative current density–voltage relationships of full-length and Δ764-TRPV1 constructs challenged with capsaicin (0.5 and 10 μm) in the whole-cell patch-clamp configuration. Bkgrd, Background currents. ***C***, Capsaicin-evoked currents in the whole-cell patch-clamp configuration of full-length and Δ764-TRPV1 constructs extracted from voltage-clamp ramp protocols at +80 mV shown in ***B***. Cap, Capsaicin; Cpz, capsazepine. ***D***, Normalized capsaicin dose–response profiles (at +80 mV) of full-length and Δ764-TRPV1 constructs. The current for each capsaicin concentration was normalized with the current at 10 μm capsaicin; *n* ≥ 4 cells per concentration. Each circle represents the mean normalized response ± SEM. Lines are Boltzmann functions fit to the data. One-way ANOVA and Bonferroni *post hoc* tests. Asterisks indicate values that are significantly different (∗∗∗*p* < 0.001); n.s. indicates values are not significantly different. ***E***, Representative current density–voltage relationships of full-length and Δ764-TRPV1 constructs challenged with pH 5.5 and capsaicin 10 μm in the whole-cell patch-clamp configuration. Bkgrd, Background currents. ***F***, pH-evoked and capsaicin-evoked currents in the whole-cell patch-clamp configuration of full-length and Δ764-TRPV1 constructs extracted from voltage-clamp ramp traces, at +80 mV, shown in ***E***. ***G***, Bar graph displaying the current magnitudes elicited by pH 5.5 and normalized by the currents elicited by 10 μm capsaicin of full-length and Δ764-TRPV1 constructs. Bars are the mean ± SEM; *n* is denoted inside the bars. Two-tailed unpaired *t* test. *p* Value is denoted above the bars.

**Figure 8.**
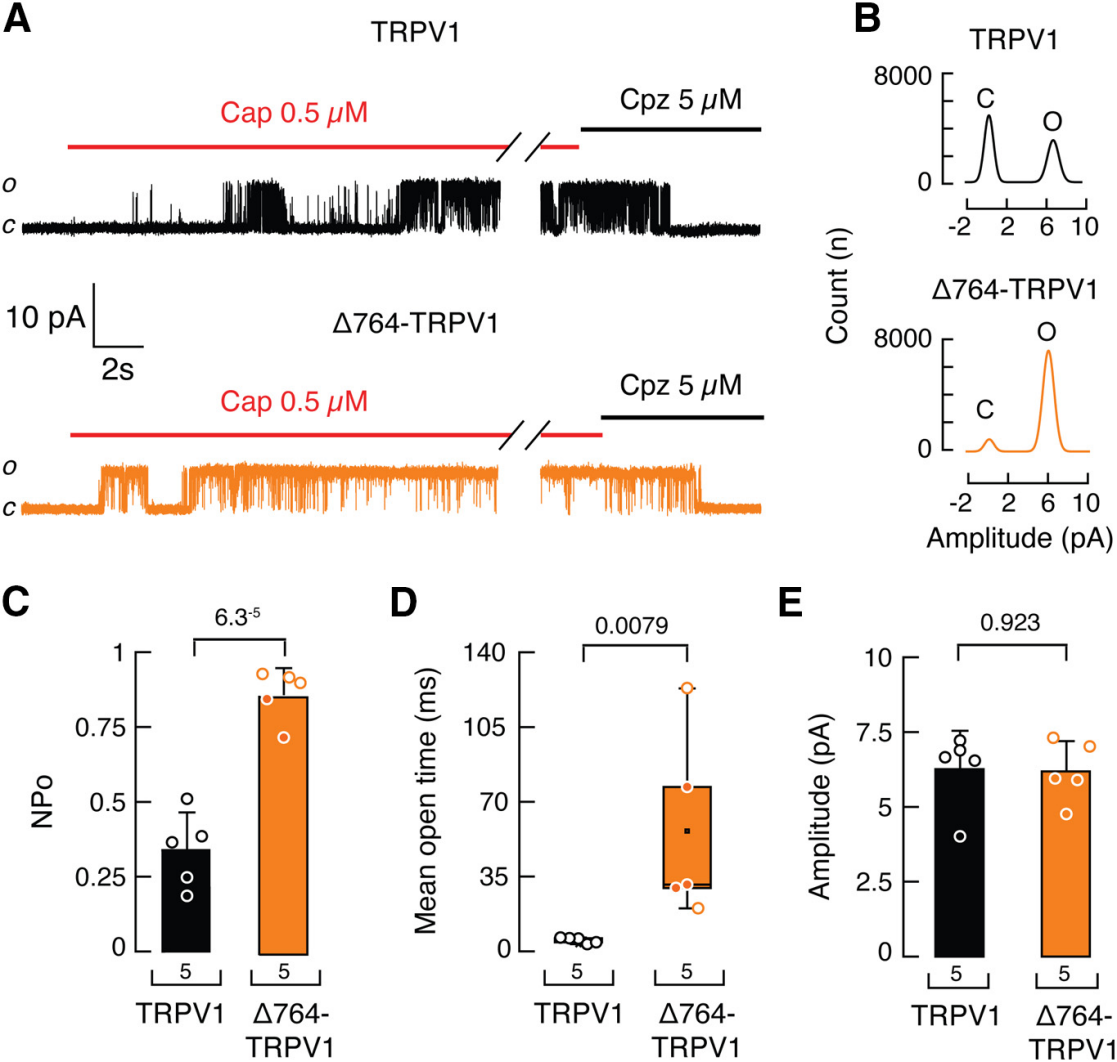
Singles-channel analyses of the Δ764-TRPV1 construct. ***A***, Representative single-channel trace recordings of full-length and Δ764-TRPV1 constructs in the inside-out patch-clamp configuration. Closed and open states are labeled *c* and *o*, respectively. Cap indicates capsaicin and Cpz indicates capsazepine. ***B***, All-point amplitude histograms of capsaicin-evoked (0.5 μm) single-channel currents from full-length and Δ764-TRPV1 constructs. Histograms were generated from recordings shown in ***A***. ***C***, Nominal open probability determined from 1 min recordings in the presence of 0.5 μm capsaicin. Bars are the mean ± SD. *n* is denoted above the *x*-axis. Two-tailed unpaired *t* test. *p* Value is denoted above bars. ***D***, Mean open times of single-channel currents elicited by capsaicin (0.5 μm) are represented in boxplots showing mean (square), median (bisecting line), bounds of box (75th to 25th percentiles), outlier range with 1.5 coefficient (whiskers), and minimum and maximum data points. Error bars represent SD. *n* is denoted above the *x*-axis. Mann–Whitney rank test for two independent groups. *p* Value is denoted above bars. ***E***, Current amplitude of single-channel openings of TRPV1 and Δ764-TRPV1 in the presence of 0.5 μm capsaicin. Bars are the mean ± SD. *n* is denoted above the *x*-axis. Two-tailed unpaired *t* test. *p* Value is denoted above the bars.

Positively charged residues within the distal C-terminal domain have been functionally identified as the phosphoinositide lipid-binding site (Fig. 7*A*). Neutralizing these charged residues enhances TRPV1 channel chemical and thermal responses (Prescott and Julius, 2003). To determine the contribution of the C-terminal domain to phosphoinositide modulation of TRPV1 *in vivo*, we constructed a rat TRPV1 mutant lacking 74 aa residues from the C terminus, including the putative phosphoinositide interaction site (Δ764-TRPV1; Fig. 7*A*). We transiently expressed and measured the function of full-length TRPV1 and Δ764-TRPV1 in HEK293 cells (Fig. 7*B–G*). Macroscopic current analyses of Δ764-TRPV1 showed an enhanced response to a low capsaicin concentration (0.5 μm) when compared with the full-length TRPV1 construct (Fig. 7*B*,*C*). The functional difference between these constructs is also underscored when comparing the response ratios between 0.5 μm and a saturating capsaicin concentration of 10 μm (Fig. 7*B*,*C*). As expected, Δ764-TRPV1 displayed a leftward shift in the capsaicin dose–response profile when compared with the full-length channel (EC_50_, 0.21 ± 0.03 vs 0.63 ± 0.02 μm, mean ± SD; Fig. 7*D*), demonstrating that this construct exhibits an enhanced response to capsaicin. Similar to capsaicin, the Δ764-TRPV1 construct displayed an enhanced response to pH 5.5 when compared with the full-length TRPV1 channel (0.55 ± 0.03 vs 0.36 ± 0.02, mean ± SEM; Fig. 7*E–G*). We also analyzed the single-channel properties of the full-length TRPV1 and Δ764-TRPV1 constructs. Removing the C-terminal region that includes the putative PIP_2_ binding site increased the steady-state NPo from 0.34 ± 0.13 to 0.86 ± 0.09 (mean ± SD; Fig. 8*A–C*). This increase in single-channel activity is the consequence of a large increase in the overall mean open time from 5.20 ± 1.34 to 56.24 ± 43.44 ms (mean ± SD; Fig. 8*D*), without any significant change in current amplitude between the constructs (Fig. 8*E*). Taken together, the Δ764-TRPV1 construct exhibits an enhanced response to capsaicin.

**Figure 9.**
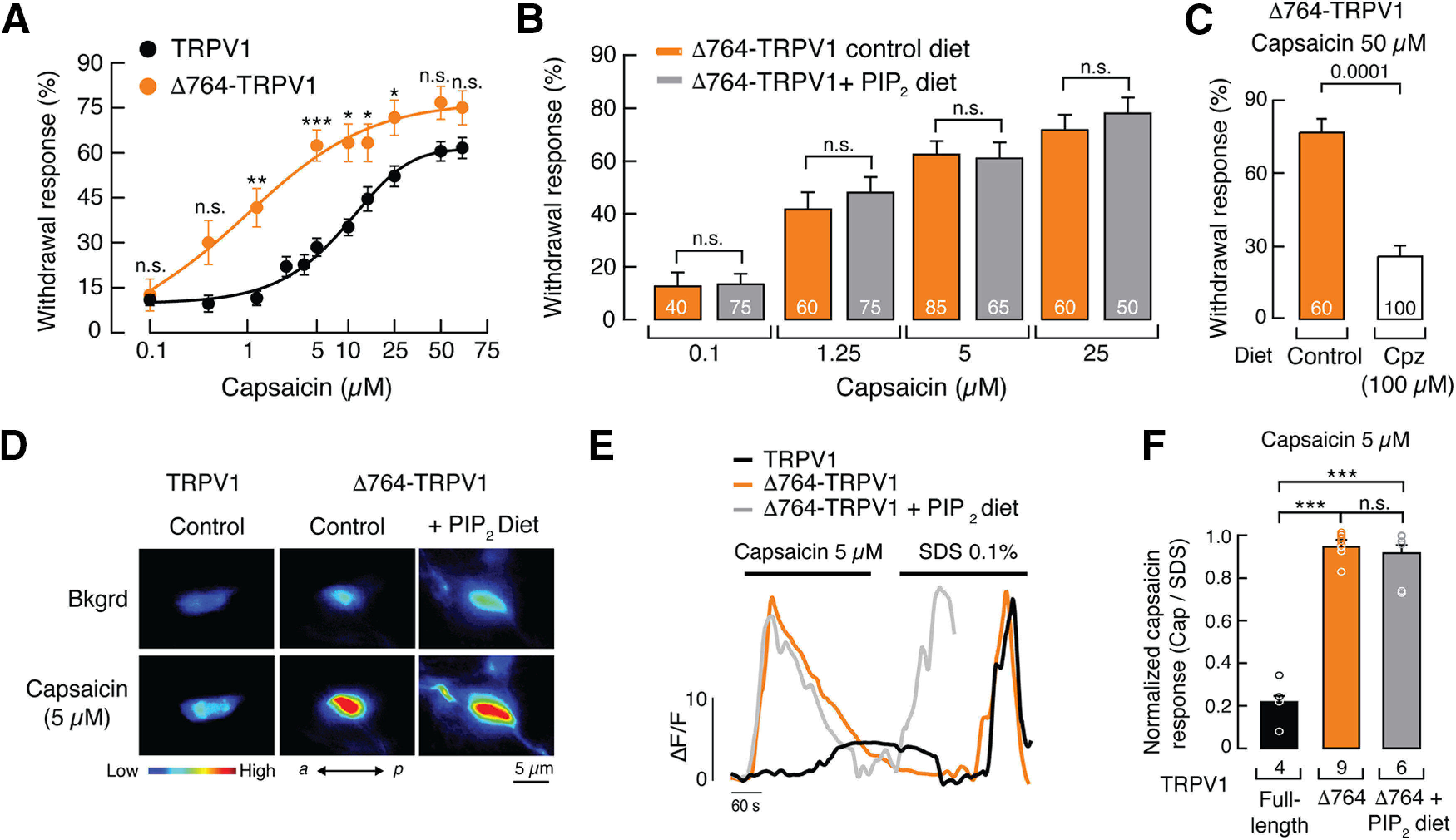
TRPV1 C-terminal domain is required for phosphoinositide lipid-mediated modulation *in vivo.* ***A***, Capsaicin dose–response profile for TRPV1 and Δ764-TRPV1 worms. Each circle represents the mean ± SEM, tested during at least three assays sessions. Lines are Boltzmann functions fit to the data. For TRPV1 worms, *n* ≥ 60; for Δ764-TRPV1 worms, *n* ≥ 40. Kruskal–Wallis and Dunn's multiple-comparisons tests. Asterisks indicate values significantly different (∗∗∗*p* < 0.001, ∗∗*p* < 0.01, and ∗*p* < 0.05); n.s. indicates values are not significantly different. ***B***, Bar graph displaying the withdrawal responses elicited by capsaicin (0.1, 1.25, 5, and 25 μm) in Δ764-TRPV1 worms fed with control or PIP_2_-supplemented diets. Bars are the mean ± SEM; the number of worms tested during at least three assay sessions are indicated inside the bars. Kruskal–Wallis and Dunn's multiple-comparisons tests. n.s. indicates values are not significantly different from the control. ***C***, Bar graphs displaying the withdrawal responses of Δ764-TRPV1 worms fed with control or capsazepine-supplemented (100 μm) diet, elicited by 50 μm capsaicin. Bars are the mean ± SEM; the number of worms tested during at least three assay sessions are indicated inside the bars. Mann–Whitney rank test for independent groups. *p* Value is denoted above the bars. ***D***, Representative micrographs of ASH neurons challenged with capsaicin (5 μm) in TRPV1 and Δ764-TRPV1-GCaMP worms fed with control or PIP_2_-supplemented (200 μm) diets. Color bar indicates the relative change in fluorescence intensity, with blue and red denoting the lowest and highest cytoplasmic Ca^2+^ (adjustment display set to 10–75), respectively. *a*, Anterior; *p*, posterior. ***E***, Representative traces corresponding to fluorescence intensity changes (Δ*F*/*F*) of individual cells shown in ***D***. ***F***, Normalized response for ASH neurons challenged with capsaicin (5 μm) in TRPV1 and Δ764-TRPV1-GCaMP worms fed with control or PIP_2_-supplemented (200 μm) diets. The capsaicin response was normalized with the response elicited by 0.1% SDS. Bars are mean ± SEM. *n* is denoted above the *x*-axis. One-way ANOVA and Tukey–Kramer multiple comparison test. Asterisks indicate values significantly different (∗∗∗*p* < 0.001); n.s. indicates values are not significantly different.

In line with our current approach, we engineered a transgenic Δ764-TRPV1 worm to test its ability to respond to capsaicin in different phosphoinositide lipid content environments. Remarkably, worms carrying the truncated version of TRPV1 displayed an enhanced response to capsaicin when compared with full-length TRPV1 worms (EC_50,_ 1.70 ± 0.45 vs 11.66 ± 3.48 μm, mean ± SD; Fig. 9*A*). If phosphoinositide lipids modulate TRPV1 function through the distal C terminus, supplementation of phosphoinositide lipids in worms lacking the phosphoinositide interaction site (Δ764-TRPV1) should not diminish their withdrawal response. Indeed, diet supplementation with PIP_2_ did not decrease the aversive behavior of Δ764-TRPV1 worms, when compared with control diet worms (Fig. 9*B*). Noteworthy, this gain-of-function mutant can be efficiently inhibited by capsazepine (Fig. 9*C*), similar to full-length TRPV1 worms (Fig. 1*E*). Hence, the Δ764-TRPV1 mutant demonstrated that, regardless of the phosphoinositide lipid content, the channel has an enhanced response compared with full-length TRPV1.

To further assess the functionality of the Δ764-TRPV1 construct *in vivo*, we measured the fluorescence intensity changes in ASH neurons of live worms after exposure to capsaicin. Capsaicin elicited robust Ca^2+^ transients in neurons of Δ764-TRPV1 worms when compared with full-length TRPV1 (Fig. 9*D–F*). Remarkably, Δ764-TRPV1 worms supplemented with PIP_2_ displayed large Ca^2+^ transients equivalent to those worms being fed with the control diet (Fig. 9*D–F*), demonstrating that this strain lacks phosphoinositide modulation. Moreover, Δ764-TRPV1 mutant results highlighted that the phosphoinositide lipid modulation is specific to TRPV1, rather than nonspecific effects elsewhere in the circuit of the worms. Interestingly, a lack of inhibition was also reported by the Rohacs laboratory when coexpressing PIP5K (i.e., the enzyme that increases PIP_2_ levels) with the TRPV1 Δ777–820 construct (Lukacs et al., 2007). Together, our results using genetic, biochemical, behavioral, and functional approaches demonstrate that phosphoinositide lipids negatively regulate TRPV1 function *in vivo* and support the idea that the phosphoinositide interaction site within the distal C-terminal domain is a key region in determining agonist response *in vivo*.

## Discussion

TRPV1 undergoes a sensitization process in which inflammatory agents enhance its sensitivity to other stimuli and, consequently, promote hyperalgesia (increased sensitivity to pain by lowering the threshold levels of nociceptors; Chuang et al., 2001; Prescott and Julius, 2003). To better understand the contribution of TRPV1 to pain hypersensitivity, it is critical to determine how molecules in the inflammatory soup alter TRPV1 function. By leveraging genetic, behavioral, biochemical, and functional approaches, we determined that phosphoinositide lipids decrease TRPV1 function *in vivo*. Three major results support our conclusion. First, we showed that chemical inhibition of an inositol monophosphatase enzyme enhanced TRPV1 function *in vivo*. Second, decreasing phosphoinositide lipid content in worms by genetic manipulation increased TRPV1-mediated aversive behavioral and neuronal responses to capsaicin, whereas worms with increased levels of phosphoinositide lipids exhibited a reduced response. Third, we demonstrated that the distal C terminus of TRPV1 is required for the phosphoinositide-mediated decreased response *in vivo*. Together, our findings support the notion that phosphoinositide lipids decrease TRPV1 response *in vivo* and that the distal C terminus is critical for fine-tuning agonist channel response.

The mechanism by which phosphoinositide lipids regulate TRPV1 function has been strongly debated. We attempt to weigh in on the debate and determine whether TRPV1 function can be modulated by phosphoinositide lipid content *in vivo* using the animal model *C. elegans*. We used the previous work of the Bargmann laboratory, who functionally expressed TRPV1 in ASH neurons of worms (Tobin et al., 2002), and the Mori laboratory, who demonstrated that worms reach adulthood after the function of a *myo*-inositol monophosphatase has been genetically impaired (Kimata et al., 2012). Using this knowledge, we engineered a transgenic worm that offers the opportunity to measure TRPV1 function while genetically reducing the phosphoinositide lipid environment. Since TRPV1 can be modulated by several phosphoinositide lipids (e.g., PI, PI3P, PI4P, PI5P, PIP_2_; Cao et al., 2013b), we avoided knocking out the function of individual kinases or phosphatases that could complicate interpretation in which channel modulation occurs because of a decrease in certain phosphoinositide lipids or increase of other upstream species.

Several regions of TRPV1 have been proposed to interact with phosphoinositides: the proximal C-terminal region (Brauchi et al., 2007; Ufret-Vincenty et al., 2011; Grycova et al., 2012; Ufret-Vincenty et al., 2015); the distal C-terminal region (Prescott and Julius, 2003; Grycova et al., 2012; Cao et al., 2013b); and close to or within the vanilloid-binding pocket (Poblete et al., 2015; Gao et al., 2016). Regardless of the proposed phosphoinositide binding sites, our *in vivo* findings demonstrate that genetically reducing phosphoinositide lipid content produces a leftward shift of the TRPV1 dose–response curve. On the contrary, supplementing phosphoinositide lipids yields a rightward shift, which is indicative of a decreased TRPV1-mediated response. Therefore, these results support the model in which phosphoinositide turnover enhances TRPV1 activity (Chuang et al., 2001; Cao et al., 2013b).

**Figure 10.**
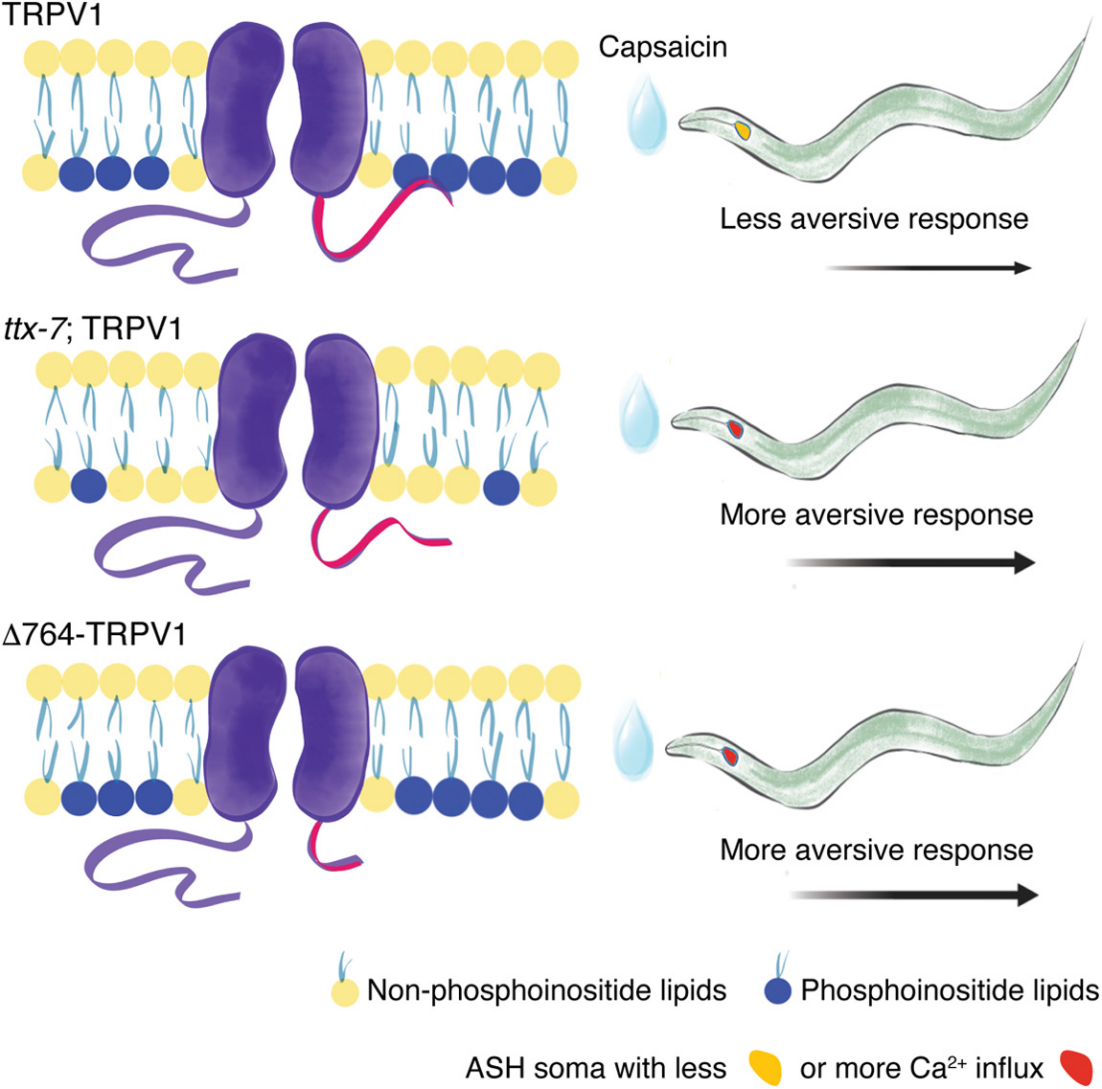
Schematic representation of the differences in phosphoinositide lipid content, behavior, and Ca^2+^ imaging in TRPV1, *ttx-7*; TRPV1, and Δ764-TRPV1 worms. Top, TRPV1 distal C terminus binds to phosphoinositide-containing membranes (in control or PIP_2_-supplemented diet conditions); in turn, capsaicin evokes Ca^2+^ influx in ASH neurons and worms display aversive responses. Middle, membranes with decreased phosphoinositide lipid content (in LiCl-supplemented diet conditions or *ttx-7* worms) reduce the likelihood of interaction of the distal C terminus with the lipids. In this condition, capsaicin evokes larger Ca^2+^ influx in ASH neurons and worms display enhanced aversive responses. Bottom, TRPV1 lacking the distal C terminus (Δ764-TRPV1) does not interact with phosphoinositide lipids. In this condition, capsaicin evokes larger Ca^2+^ influx in ASH neurons and worms display enhanced aversive responses, regardless of the phosphoinositide lipid content.

Our *in vivo* approach does not directly address whether the TRPV1 C terminus physically interacts with the phosphoinositide-containing membrane. However, a previous result using TRPV1/DGS-NTA-containing liposomes, with and without nickel, supported a direct interaction between the distal TRPV1 C terminus and membrane lipids (Cao et al., 2013b). Importantly, the cryo-EM structures of TRPV1 lack 74 residues of the C terminus. Hence, there is no direct structural evidence of the C-terminal/membrane interaction (Liao et al., 2013; Cao et al., 2013a; Gao et al., 2016). The diagram in Figure 10 summarizes our *in vivo* analyses in light of the functional TRPV1/DGS-NTA-containing liposome results. If the C-terminal/membrane interaction is circumvented by decreasing phosphoinositide lipid content (*ttx-7*; TRPV1 worms) or by removing the putative protein–membrane interaction site (Δ764-TRPV1), TRPV1 would increase neuronal activation and enhance agonist response (Fig. 10, middle, bottom). Of note, we found that the increased response of Δ764-TRPV1 to capsaicin stems from a large increase in the mean open time, when compared with the full-length channel. On the other hand, an intact C-terminal/membrane interaction reduces TRPV1 agonist response (Fig. 10, top). Interestingly, the enhanced withdrawal behavior of Δ764-TRPV1 worms resembles the increased agonist response reported in the TRPV1 C-terminal splice-variant expressed in the trigeminal ganglia of vampire bats (Gracheva et al., 2011).

Members of the TRPV subfamily share the ability to be modulated by phosphoinositide lipids. For instance, previous works have shown that phosphoinositides decrease TRPV1, TRPV3, and TRPV4 channel activity (Doerner et al., 2011; Cao et al., 2013b; Takahashi et al., 2014; Rohacs, 2015; Harraz et al., 2018), whereas other works propose a potentiation effect on TRPV1, TRPV2, TRPV4, TRPV5, and TRPV6 channel function (Lee et al., 2005; Rohács et al., 2005; Thyagarajan et al., 2008; Mercado et al., 2010; Garcia-Elias et al., 2013; Rohacs, 2015; Hughes et al., 2018). Based on these studies, it becomes clear that there is no general mechanism for the effect that phosphoinositide lipids exert on members of the TRPV channel subfamily. Some TRPVs seem to be negatively regulated by phosphoinositides, while others are positively modulated, and in other cases there is not yet a consensus. In our opinion, this is likely because of the difficulty of manipulating and measuring cellular lipid content.

Our experimental approach demonstrates that phosphoinositide lipid content can be manipulated in worms via the TTX-7 enzyme and diet supplementation. *C. elegans* offers a repertoire of genetic tools for manipulating lipid content; therefore, future experiments using this model could help to determine the specific effect of individual phosphoinositide lipid species on channel function. Beyond TRP channels, this approach would be useful for understanding how membrane lipids modulate other receptor-operated ion channels.
